# Supplementary material for: Neuromorphic antennal sensory system
Source: Nat Commun. 2024 Mar 7;15:2109. doi: 10.1038/s41467-024-46393-7 (PMC10920631; doi:10.1038/s41467-024-46393-7)
Supplement: Supplementary file 1 — Supplementary Information [file 41467_2024_46393_MOESM1_ESM.pdf]

Neuromorphic antennal sensory system

Chengpeng Jiang[1,2], Honghuan Xu[1,2], Lu Yang[1,2], Jiaqi Liu[1,2], Yue Li[1,2], Kuniharu Takei[3*], Wentao Xu[1,2*]

[1] Institute of Photoelectronic Thin Film Devices and Technology, Key Laboratory of Photoelectronic Thin Film Devices and Technology of Tianjin, College of Electronic Information and Optical Engineering, Engineering Research Center of Thin Film Photoelectronic Technology of Ministry of Education, Smart Sensing Interdisciplinary Science Center, Nankai University, China

[2] Shenzhen Research Institute of Nankai University, China

[3] Graduate School of Information Science and Technology, Hokkaido University, Japan

* Corresponding authors. Email: takei@ist.hokudai.ac.jp; wentao@nankai.edu.cn

Supplementary Note 1

Details on signal acquisition and signal processing

In the electronic antennal nerve system, the signals from the two sensors are acquired using a microcontroller-based peripheral circuit. This peripheral circuit converts the sensor's piezoelectric signal into a voltage signal and then encodes this sensory signal into fast-adapting and slowly-adapting spike trains. The acquiring frequency of the sensor signal is typically set to 200 Hz or 600 Hz, depending on the requirements of different sensation tasks. Note that the acquiring frequency of the sensor signal affects its temporal resolution, and a higher acquiring frequency can better exhibit the oscillating or changing behaviors of the sensor signal, especially during the vibrotactile sensation process. The sensor signals are transformed into two pairs of SA and FA spikes. The instantaneous firing frequency of the SA and FA spikes is typically set to 50 Hz during the spike-encoding process, considering the frequency-dependent synaptic characteristics of the artificial synaptic device. The pairwise SA spikes are sent to the SA device, and similarly, the pairwise FA spikes are forwarded to the FA device. The synaptic currents of the SA and FA devices are recorded at 100 Hz.

Supplementary Note 2

Sensing mechanism of the electronic-antennae sensor

The sensing mechanism of the sensor is based on the piezoelectric effect and magnetic interaction. The first figure below illustrates the working mechanism for tactile sensing. The sensing material in our flexible sensor is piezoelectric materials (PVDF), and this material can generate electric charge in response to applied mechanical force or stress. This electromechanical energy conversion is the piezoelectric effect. In the absence of external force, the piezoelectric film of the flexible sensor does not deform and no electric signal is generated. Upon the application of an external tactile force (denoted as $F_{tactile}$), the flexible sensor is bent, inducing lateral strain of the piezoelectric material and a decrease of its polarization. This deformation triggers a potential difference (built-in electric field), and thus an electric current is generated ($I > 0$). When the bending-induced strain is released, an electric current with opposite direction ($I < 0$) is generated.

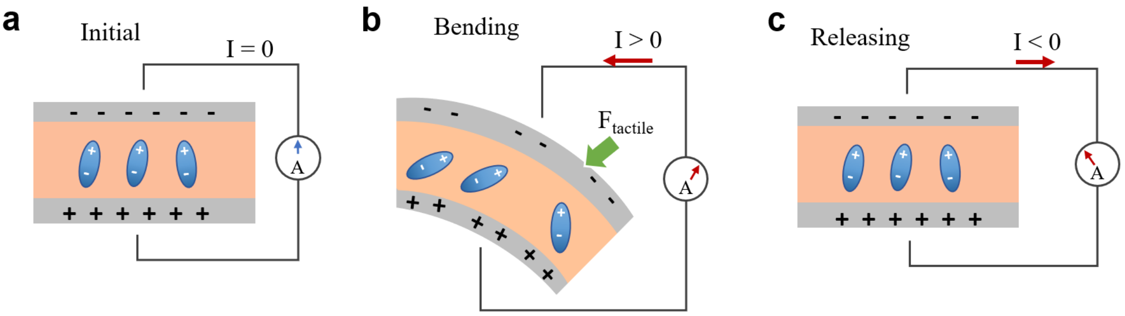

Working mechanism of the sensor for tactile sensing.

The second figure below illustrates the working mechanism for magnetic sensing. Our flexible sensor can be modeled as a flexible beam loaded with a magnetic appendage (NdFeB) that was axially magnetized. In the absence of magnetic field, the flexible sensor is not deformed. When a magnetic or ferromagnetic object approaches to the flexible sensor, the interaction between the magnetic appendage and the object is enhanced, manifested by an increasing magnetic interaction force (denoted as $F_{magnetic}$). Consequently, the flexible sensor is deformed due to the magnetic interaction force, and the piezoelectric material attached to the flexible film generates an electric current ($I < 0$) during this deformation. Upon the retraction of the magnetic/ferromagnetic object, the magnetic interaction force is reduced and the flexible sensor is gradually recovered to its initial state. This recovery process results in an opposite current ($I > 0$).

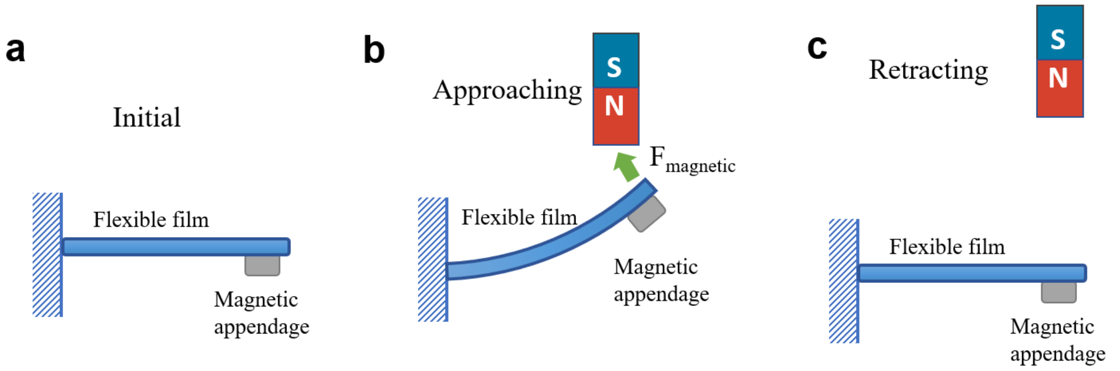

Working mechanism of the sensor for magnetic sensing.

Supplementary Note 3

Modeling of the electronic-antennae sensor

The antennae structure of the electronic-antennae sensor can be modeled as a mass-less cantilever beam with a rectangular cross-section. Fixed-end and free-end conditions are applied to the bottom and the top of this cantilever beam, which is illustrated in the following figure:

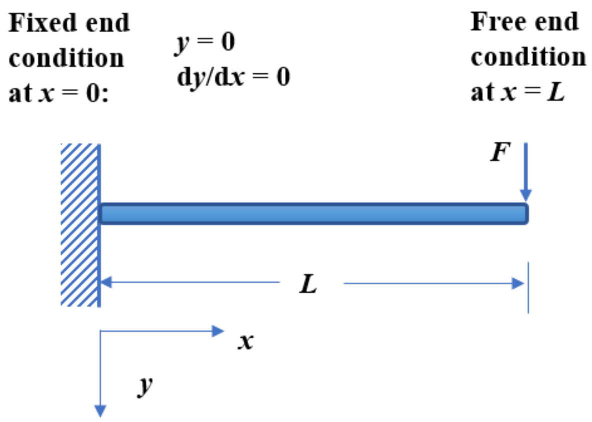

The length, thickness, width, elastic modulus (Young's modulus), moment of inertia, and spring constant of this cantilever beam are defined as *L, t, w, E, I,* and *k,* respectively. Specifically, the thickness direction is along the displacement direction of the beam, while the width direction is perpendicular to the deflection direction of the cantilever beam. From beam theory, the relation between beam deflection $y(x)$ at a distance of x and applied load F is given below:

$$y(x) = \frac{F}{6EI}(3x^2L - x^3)$$

$$I = \frac{wt^3}{12}$$

At the free end of the cantilever beam, the tip deflection $y(L)$ is given as:

$$y(L) = \frac{FL^3}{3EI}$$

The above equation suggests that the relationship between applied force and beam deflection is linear for all thicknesses.

Furthermore, the cantilever beam can be represented by an equivalent spring, and its effective spring constant (k) can be derived as follows:

$$k = \frac{Ewt^3}{4L^3}$$

This equation reveals that the spring constant (transverse stiffness) is dependent on the geometry of the cantilever beam, and the spring constant versus cantilever thickness exhibits a power-law relationship. In our electronic-antennae sensor, the length (L) is typically fixed at 2 cm considering the requirements of profile classification and non-contact sensation, the width is set to 3 mm, which equals the width of piezoelectric film, and the thickness of the sensor (the thickness of the PET plastic substrate) can be changed to adjust the spring constant. The optimized thickness of the polyimide substrate is 180 μm.

The final form of the electronic antennae sensor has a magnetic appendage mounted on the tip. The final sensor can be thus modeled as a mass-less beam carrying a mass (M) at its free end, and bending vibration can be induced by giving an initial displacement at the free end. Free vibration of the cantilever beam with a lumped mass at the free end is illustrated in the following figure:

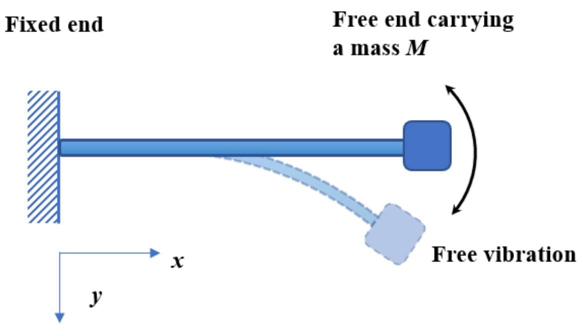

The natural angular frequency (ω) and the natural standard frequency (f) of this system under no damping can be derived as:

$$\omega = \sqrt{\frac{k}{m}} = \sqrt{\frac{3EI}{mL^3}}$$

$$f = \sqrt{\omega/2\pi}$$

Therefore, the undamped vibration of this system is simple harmonic oscillation. The vibration frequency of this system (the electronic-antennae sensor) is related to the spring constant of the cantilever beam (the plastic substrate) and the mass of the loaded object (the magnetic appendage).

Supplementary Note 4
Pseudocode for the spike-encoding strategy

In the electronic antennal nerve system, the spike-encoding function is realized by programming the microcontroller using simple software code. The pseudocode for implementing the spike-encoding strategy is shown below:

```
Define the threshold for SA signal as SA_th1, for example, 15;
Define the threshold for FA signal as FA_th1, for example, 5;
Define the default output of the sensor under no stimuli as SENS_DEF;
Define the maximum number of the data points read from the sensor as N;

Main Loop of spike encoding function ( )
{
                Define two counters as counter1 = 0, counter2 = 0;
                Define Fast- and Slow-Adapting spikes as FA[i] and SA[i];
                Define sensor signal as SENS[i];
                for (i=2; i < N; i=i+1)
                {
                        Read sensor signal and store it to SENS[i];
                        if (counter1 == 0)
                        {
                                if ( abs(SENS[i] - SENS[i-2]) < FA_th1 )
                                {
                                        counter1 = 1; FA[i] = 0, generate FA spike of “0V”;
                                }
                                else
                                {
                                        counter1 = 1; FA[i] = 5, generate FA spike of “5V”;
                                }
                        }
                        else
                        {
                                counter1 --; FA[i] = 0, generate FA spike of “0V”;
                        }
                        if (counter2 == 0)
                        {
                                if ( abs(SENS[i] - SENS_DEF) < SA_th1 )
                                {
                                        counter2 = 1; SA[i] = 0, generate SA spike of “0V”;
                                }
                                else
                                {
                                        counter2 = 1; SA[i] = 5, generate SA spike of “5V”;
                                }
                        }
                        else
                        {
                                counter2 --; SA[i] = 0, generate SA spike of “0V”;
                        }
}
```

In this code, the spike encoding function starts with reading the current value of the sensor signal. Then, the current sensor value is compared with the previous sensor value. If the absolute difference between them is larger than the predefined FA threshold, an FA pulse is generated. Subsequently, the current sensor value is compared with the default sensor value, and if the absolute difference is greater than the predefined SA threshold, an SA pulse is generated. In this way, two pairwise sets of FA and SA spike trains are simultaneously derived from the signal of one sensor, achieving the spike-encoding function. The spike trains are then sent to the artificial synaptic device for spatiotemporal processing. Besides, to improve the memory and retention behavior of the artificial synaptic device, sustaining spikes with reduced amplitude (0~1.5 V) are added to the firing sequence of the SA or FA spike trains. As for accomplishing different tasks of mechanosensation and magnetosensation, the sampling rate of sensor signals and the instantaneous firing rate of the SA and FA spike trains were adjusted accordingly. For example, the instantaneous firing rate of SA and FA spike trains was typically set to 50 Hz while the sensor signal was sampled at 200 Hz.

Supplementary Note 5
Magnetic interaction force during magneto-sensation

Calculating the magnetic field and the magnetic interaction force is complex, as it depends on the magnetization, geometry, orientation, and separation of the magnet and the object.
For simplicity, the magnetic field produced by a uniformly magnetized magnet (in our case, an axially magnetized cylindrical magnet with a radius of R and thickness of $2b$) can be approximated to the field generated by a steady current (I) flowing in stacked current loops (or a solenoid) with the same geometry, as shown in the following figure.[1,2]

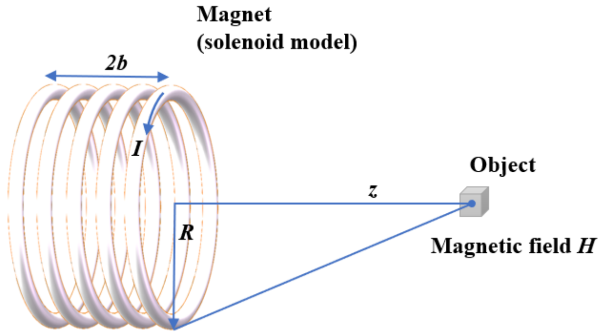

Based on the Biot-Savart law, the magnetic field (B) of this solenoid model (total turns of N) at the origin of the loop center (maximum value) and an arbitrary point along the axial direction (Z axis) can be derived as:

$$B_{\max|z=0} = \mu_0 NI \frac{1}{2\sqrt{b^2 + R^2}}$$

$$B_z = B_{\max} \cdot \left[\frac{z+b}{\sqrt{(z+b)^2 + R^2}} - \frac{z-b}{\sqrt{(z-b)^2 + R^2}}\right] / \left[\frac{2b}{\sqrt{b^2 + R^2}}\right]$$

where μ_0 is the permeability for empty space. Therefore, the magnetic field at different distance can be obtained theoretically by calculation.

Experimentally, we further measured the magnetic field generated by a cylindrical magnet (diameter 4 cm, thickness 1 cm) using a gaussmeter at different distances (ranging from 0 cm to 7 cm). Thus, the theoretical values and the measured values of the magnet field can be compared, as presented in the following figure:

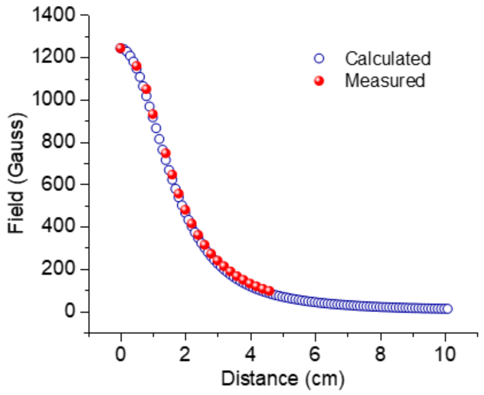

The result shows that the magnetic field from the cylindrical magnet is highly distance-dependent, and it decays from the maximum values of ~1200 Gauss to ~200 Gauss at a distance of 3 cm. For magneto-sensation tasks, the distance between the sensor's tip and the magnetic object's surface was restricted to less than 3 cm so that the magnetic field exerted on the sensor is high enough to trigger sufficient mechanical deflection of the antennae structure in the sensor.

Supplementary Note 6
Comparison with Existing Bioinspired Flexible Sensors

We systematically reviewed recent articles on bioinspired flexible sensors, focusing on tactile and magnetic sensation.[33-39] These sensors are mostly inspired by the tactile sensory organ of mammalian skin, often featuring a multilayered planar or out-of-plane hairy structure. Although some flexible sensors in these works employ a three-dimensional structure to enable adaptive sensation, their fabrication involves high-cost, complicated manufacturing processes such as photolithography and micropatterning. Moreover, despite the utilization of functional materials with excellent sensing capabilities, such as carbon nanotubes, nanofibers, triboelectric material, and piezoelectric materials, the tactile sensing functions are generally limited to pressure, temperature, friction, and bending detection. Vibrational and non-contact tactile sensation (such as magnetically responsive tactile perception) are rarely explored in these bioinspired sensors. Regarding sensory processing and sensory encoding, the state-of-the-art flexible sensors recently reported in the literature (e-skin, magnetosensitive e-skins, biomimetic whisker) can achieve high sensitivity and fine spatial resolution in detecting sensory stimuli. However, in most cases, the acquired sensing signals are processed in digital form using external computational and memory resources (for instance, computer). Some works employ spike-coding method for signal processing, but sensory processing is typically implemented through machine-learning algorithm or the Fourier transform method. Therefore, these reported flexible sensors, along with their sensory systems, have made limited attempts to implement bioinspired signal encoding and processing using biologically plausible methods (e.g., synaptic processing) or brain-inspired hardware (e.g., synaptic transistors).

In comparison with currently reported bioinspired flexible sensors, our electronic antennae sensor exhibits unique characteristics and advantages as outlined below.

(1) Sensor Structure and Function: our electronic antennae sensor features segmented, flexible, three-dimensional structures and multifunctional sensing capabilities, including vibrotactile and magneto perception. By emulating the antennal sensory system of insects, our sensor breaks the design constraints of current electronic skin sensors that are inspired by skin. In addition, our sensor offers various operational modes for detecting stimuli including deflection, vibration, and magnetic field with low detection limit, thus enabling advanced applications such as profile classification, texture discrimination, Braille code recognition, material classification, magnetic navigation, and touchless interfacing. These versatile functions and applications are hard to achieve using other bioinspired tactile sensors.

(2) Signal Encoding and Processing: our sensory system employs spike-based signal encoding strategies (fast-adapting and slowly-adapting coding) inspired by mechanoreceptors. Furthermore, our sensory system comprising electronic antennae sensors and synaptic devices creates a 2-by-2 receptor-neuron network that emulates the “labeled-line” model of the biological receptor-neuron pathway. This design facilitates the separate processing of static and dynamic signals. Achieving device-level cognitive perception of spatiotemporal sensory information in a parallel, energy-efficient manner, our system excels in synaptic processing with neuromorphic intelligence. Additionally, important neuronal functions, including spatiotemporal integration, multimodal sensory processing, and controllable sensory memory, are realized in the synaptic device. These advancements contribute to the achievement of vibrotactile and magneto perception, which are scarcely attempted in other artificial sensory systems. Notably, spike encoding, synaptic processing, and neuronal functions are implemented in the neuromorphic hardware and the microcontroller, making our system more suitable for wearable applications compared to other bioinspired sensory systems relying on external computing or memory resources (such as CPU, GPU, or DSP processors).

Supplementary Note 7

Evaluation and improvement of device-to-device variations

Device-to-Device Variation: First of all, device-to-device variation was evaluated. A total of 12 synaptic transistors, fabricated on a flexible substrate, were tested by applying voltage spikes (+5V sensory spikes followed by +1V sustaining spikes) and recording the synaptic current. The important parameter of memory retention behavior, which is essential for sensory processing, was calculated here as the ratio of synaptic current at $t = 20$ s (acquired after the stop of spike stimulation) to the resting current (acquired before spike stimulation). Figure S14 (a) illustrates the device-to-device variations of memory retention behaviors for all 12 devices, indicating a variation of approximately 7.1%.

Analysis of Contributing Factors: We then individually analyze the factors that influence device performance and device-to-device variations. Firstly, our previous results on material characterization and device fabrication shows that morphology of the metal-oxide semiconducting thin film is critical for achieving desirable transistor properties of the device. The metal-oxide semiconducting film with too thin thickness or with too many defects will result in discontinuous or pin hole structures, which are detrimental to the formation of a uniform semiconducting channel. Our optimized fabrication process produces a smooth and continuous metal-oxide film by treating the substrate using UV-Ozone (10 min), which increase the surface energy and wetting capability of the substrate, and then annealing the spin-coated film at elevated temperature (300 °C for two hours), which effectively transform the precursor material into metal oxide material. These steps ensure both uniform morphology and semiconducting properties of the metal-oxide film. Secondly, the distribution and properties of the nanoflakes is also important for improving the transistor and synaptic properties of the device. Note that the fabrication process, optimized for the adsorption of nanoflakes, involves purifying the nanoflake solution and controlling the conditions of the liquid-phase adsorption. This results in highly-crystalline nanoflakes densely distributed without obviously aggregation at the surface of metal-oxide film. The distribution of the nanoflakes affect the device performance. When the nanoflakes are insufficiently purified, large aggregation of the nanoflakes with poor distribution will appear and compromise the electrostatic gating effect, considering that the 2H-phase MoS_2 nanoflakes prepared by liquid-phase exfoliation is wide band-gap semiconducting material with organic ligands. Moreover, a performance comparison of the synaptic devices fabricated without and with nanoflakes demonstrates that high-density nanoflakes enhance the device performance by increasing the on-off ratio (from 7.5 to 84.5), suppressing the off-state current (from 3.05 to 0.15 μA), increasing the linearity of potentiation behavior, and improving the signal-to-noise ratio of synaptic response. These enhancements can be attributed to the charge trapping effect of the nanoflakes located at the interface between semiconducting metal-oxide film and the alginate ion gel. Additionally, shifts in the threshold voltage (from 1.0 to 2.4 V) were observed in the transfer curve, and it can be related to the built-in field arising from carrier trapping. Thirdly, the properties of the ion gel are critical for efficient electrostatic gating of the synaptic device. The frequency-dependent specific capacitance of the ion gel film is confirmed, exhibiting a large specific capacitance of ~1.5 μF cm^{-2} at a frequency of 1 kHz. This large capacitance arises from the formation of electrical double layers (EDL) at the interface. The capacitance of the ion gel film is strongly dependent on frequency (up to 10 kHz), since the mobilities of the ions (in our case the ions are mostly protons with high mobility) limit the polarization response time. The estimated formation time of the double-layer capacitances for the ion-gel electrolyte is in the order of 10 μs, which allows synaptic response under stimuli of voltage spikes close to 100 kHz. The large specific capacitance of the ion gel and the rapid formation of the EDL layer attributed to the high mobility of the ion can ensure effective electrostatic gating in the synaptic transistor. Besides, the ion gel exhibits high mechanical flexibility, good ambient stability, and large resistance, making it beneficial for flexible electronics applications. Regarding variations of device performance, it is noteworthy that our synaptic device, prepared on a flexible substrate using solution-processable methods, inherently exhibits higher device-to-device variation than other synaptic devices prepared on silicon substrates

through physical or chemical deposition methods.

Mitigation Strategy: Furthermore, we developed an experimental strategy to effectively mitigate the device-to-device variations. As shown in Figure 3h, we have verified that the memory retention behavior and sensory memory of the synaptic device can be modulated by applying sustaining spikes with small amplitude (ranging from 0 to 1.5 V) after the stimuli of sensory spikes (5 V). For precise regulation of memory retention behavior, here we finely adjust the amplitude of the sustaining spike using a small step size (minimum step size 0.05V) while recording the synaptic current from the device. The results shown in Figure S14 (b) reveal that multi-level memory states of the synaptic current are achieved by finely tuning the amplitude of the sustaining spikes. Therefore, memory retention behavior for each synaptic device can be individually regulated by appropriately setting the sustaining spike's amplitude to ensure similar retention behavior. To implement this strategy, we experimentally modulated the memory retention behaviors of all 12 devices by adjusting the amplitude of the sustaining spike (minimum step size 0.05V), and the device-to-device variation is evaluated again. As presented in Figure S14 (c), the value of device-to-device variation is reduced from 7.1% to 2.6%. This strategy of adjusting the sustaining spike's amplitude effectively mitigates the issue of device-to-device variation by enabling manual control of device memory. Moreover, this strategy can ensure good stability and repeatability across multiple experiments or across different devices.

Influence on Classification Accuracy: Finally, we investigate the influence of the device-to-device variation on classification accuracy. In our initial setup, as shown in Figure 4e, the recognition task of chess profile classification was executed using identical synaptic devices, yielding a recognition accuracy of 0.916, and this case did not consider the device-to-device variation. Taking account of the device-to-device variation, the classification task was repeated again using different synaptic devices. The strategy of adjusting the sustaining spike's amplitude as mentioned above was employed to ensure similar memory retention behaviors across diverse devices. As a result, the recognition accuracy for chess profile classification, while considering the device-to-device variation (2.6%), slightly decreased from 0.916 to 0.892. Therefore, the device-to-device variation of the device memory may diminish the classification accuracy of tactile recognition. Nevertheless, the strategy of adjusting the sustaining spike's amplitude we developed here can mitigate such influence by reducing the device-to-device variation.

Supplementary Note 8

The correlation coefficient of pairwise spike signals

The correlation coefficient of two pairwise spikes emitted from neurons 1 and 2 in a given period of T was calculated using the following equation:[3]

$$\rho_{12} = \frac{\mathrm{Cov}[n_1(T), n_2(T)]}{\sqrt{\mathrm{Var}[n_1(T), n_1(T)] \cdot \mathrm{Var}[n_2(T), n_2(T)]}}$$

where $n_1(T)$ and $n_2(T)$ represent the two spike trains fired from neurons 1 and 2, respectively, and ρ_{12} measures the strength of pairwise correlation. A correlation coefficient of 1 means that the two spike trains are identical and correlated, while a correlation coefficient of 0 signified that the two spike trains are independent.

Supplementary Note 9

Training and inferring procedures for recognition and classification tasks

Training and inferring procedures were performed during recognition and classification tasks, including chess profile and Braille code classification. The training procedure was executed to obtain the decision boundaries for classification. By completing the tactile sensation operations, hardware outputs involving the mean firing rates (f_{SA}, f_{FA}) of SA and FA spikes and the ending values (I_{SA}, I_{FA}) of synaptic current from SA and FA devices were acquired in terms of the type of test objects, such as chess pieces or Braille patterns (type of the object was known). The ending value of synaptic current was obtained at the end of a sensory event when the sensory spikes stopped generating. For instance, if a sensory event ends at $t = t_0$, the synaptic current of the SA device and FA device (denoted by I_{SA} and I_{FA}) at t_0 is recorded. The mean firing rate of encoded spikes was derived by averaging the number of pairwise spikes over the duration of a sensory event. For example, the mean firing rate of FA spikes (denoted by f_{FA}) is calculated by counting the average number of FA1 and FA2 spikes (denoted by N_{FA1}, N_{FA2}) and then dividing this average spike number over the event duration (t_{event}), i.e., $f_{FA} = (N_{FA1} + N_{FA2}) / 2 / t_{event}$. In this way, these hardware outputs were obtained in an event-based manner, and different levels of the hardware output correspond to different classes of the test object. The decision boundary between various classes was set as the midpoint of the neighboring levels of the hardware outputs. Following this, an inferring procedure was conducted to predict the type of random objects. Firstly, hardware outputs (f_{SA}, f_{FA}; I_{SA}, I_{FA}) were recorded for each object through tactile sensation. Then, a decision-tree method that compares the hardware outputs and their decision boundaries was adopted to recognize and classify the object type. All the operations were implemented in the neuromorphic system at the hardware level, which means that the sensation function, the synaptic processing, and the training/inferring procedures were performed using the electronic-antennae sensor, the artificial synaptic device, and the microcontroller-based peripheral circuit. Finally, the recognition/classification accuracy was derived by calculating the ratio of correct predictions to the total predictions.

Supplementary Note 10

Influence of scanning speed on tactile recognition

We have performed additional experiments to systematically investigate the influence of scanning speed on the frequency and amplitude of the sensor signal. Surface patterns with periodic ridges were used as the object of interest for tactile perception. A total of four surface patterns with different ridge widths (0.5, 0.7, 0.9, 1.1 mm) were laterally scanned by the flexible sensor at various scanning speeds (2, 4, 6, 8 mm s^{-1}).

Analysis of Sensor Signal: The obtained sensor signals are presented in Figure S20 (a)-(d). It can be observed from the experimental results that as the scanning speed increases, the frequency of the sensor signal increases correspondingly, while the intensity (amplitude) of the signal does not change obviously. A closer examination of the sensor signal reveals that lateral sliding at various scanning speeds all causes vibrations in the sensor, and each stripe of the surface pattern triggers a tactile signal with oscillation behavior.

Analysis of Device Output: Moreover, the output of the fast-adapting (FA) device during surface scanning process is presented in Figure S20 (e), and the device output exhibits stepwise spiking, corresponding to the periodic stripes of the sample surface. The time interval between each spiking event is crucial for matching sensory memory and facilitating sensory processing. It can be thus inferred that the scanning speed, affecting the timing of spiking events in device output, needs to be controlled within a reasonable range.

Impact of Scanning Speed: We further examine the effect of scanning speed on the recognition accuracy in a tactile perception task. Figure S20 (f) shows the relationship between the scanning speed of the sensor and the accuracy of surface pattern recognition. The classification accuracy is the highest under the scanning speed of 6 mm s^{-1}, meaning that 6 mm s^{-1} is the optimal scanning speed for the surface pattern recognition task. Note that in our original setup, the scanning speed of the sensor was chosen as 5 mm s^{-1}, which is close to the optimal value, and thus the recognition experiments we performed are reliable. A slow scanning speed will increase the time interval between tactile events, and this may cause memory loss and reduce recognition accuracy significantly. Conversely, a fast scanning speed may induce the “shadow effect”, similar to the case when an atomic force microscopy tip scans quickly across a sample surface, and consequently the sensor signal cannot resolve the surface patterns, leading to reduced recognition accuracy. The relationship between scanning speed and recognition accuracy highlights the significance of choosing an appropriate scanning speed.

Task-Specific Variations in Optimal Scanning Speed: In profile classification task, the shape of the chess piece needs to be identified, given that the chess piece has smooth surface but large irregular contour. The lateral scanning process mainly induces the significant deflection (typical deflection >3 mm) of the flexible sensor, resulting in the prominent response in the SA sensory spikes. A relatively slow scanning speed can potentially ensure the gradual deformation of the flexible sensor and the effective accumulation of synaptic current from the SA device. In surface/texture classification task, the morphology of the surface needs to be perceived, given that the sample surface is flat but features small periodic stripes or textures. The lateral scanning operation mainly induces repeated vibrations of the flexible sensor (typical deflection <0.5 mm), leading to the intense response in the FA sensory spikes. A relatively fast scanning speed can ensure the intense vibration of the sensor tip during scanning across the pattern or texture, resulting in large output from the FA device.

For human tactile exploration experiment, the preferred scanning speed of the volunteer’s finger was slower when identifying shapes of the chess piece, in contrast to the faster scanning speed employed when identifying surface patterns. This discrepancy suggests that human employs distinct scanning speeds of finger motion during surface recognition task and profile recognition task to achieve optimal perceptual performance. For our neuromorphic system, the optimal scanning speed of the sensor also varies with perception tasks. The variation is attributed to the distinct goals of surface recognition and profile recognition, aimed at identifying surface texture and shape morphology, respectively. Texture information is obtained through the vibration of

the flexible sensor, while shape information is acquired by the deformation of the flexible sensor.

In conclusion, the scanning speed of the sensor during tactile perception has a great impact on the frequency of the sensor signal, while it has trivial influence on the amplitude. Choosing an appropriate scanning speed of the sensor is crucial for achieving reliable tactile recognition, considering its effects on sensory memory and processing. In our specific case, the scanning speed of the sensor during tactile perception was appropriately set to guarantee reliable tactile recognition results.

Supplementary Note 11

Improvement of recognition accuracy

To enhance the recognition accuracy for objects and textures as shown in Figure S24 (a)-(b), we propose two experimental methods. The first method is to appropriately increase the pre-deflection applied to the tip of the electronic antennae sensor during its contact with the object of interest, thereby increasing the contact depth between the sensor tip and the target object. Figure S24 (c)-(d) shows the sensor signal for chess profile classification (King) obtained at contact depth (maximum value) of 3 mm and 3.5 mm, demonstrating that an increased contact depth leads to a higher amplitude of the sensor signal (spiking intensity was enhanced). Similarly, Figure 24 (e)-(f) exhibits the sensor signal for surface texture classification (M3 and M4 samples) acquired at contact depth (maximum value) of 1 mm and 1.5 mm, revealing that an enlarged contact depth results in higher intensity and finer resolution of the surface texture information. These experimental results confirm that appropriately increasing the contact depth may improve the quality of the sensor signal, thereby potentially improving the recognition accuracy of perception tasks. It is noteworthy that increasing the contact depth will increase the contact force exerted on the sample surface, potentially causing indentation of the surface material and large deflection of the sensor tip. Therefore, the contact depth should be controlled within an appropriate range (<6 mm in our setup). The second method is to employ firing rate coding for the sensory spikes. In our initial setup, the sensor signal was encoded into fast-adapting (FA) and slowly-adapting (SA) sensory spikes with a fixed frequency, meaning that a single threshold value was used during the encoding process. By employing firing rate coding during the encoding process, multiple threshold values can be utilized to generate sensory spikes with various frequencies, thereby increasing the efficiency of information encoding. This firing rate coding method, which generates sensory spikes with rate-modulated information, holds potential to further improve the recognition accuracy of perception tasks.

Supplementary Note 12
Comparison with bio-inspired visual sensory systems

We compare our work with recent advancements in insect-inspired and bio-inspired artificial sensory systems.[26-32] Typically, the majority of cutting-edge artificial sensory systems are designed as artificial visual sensory systems that are inspired by mammalian eyes or insect compound eyes, wherein the sensory input is limited to visual signals. These systems often utilize planar structures on rigid silicon or spherical structures on flexible substrates, and the lack of three-dimensional structures with flexible or adaptive designs poses a limitation. Moreover, the fabrication of these systems involves chemical or physical deposition and other complicated manufacturing procedures to grow functional materials, so the fabrication cost is high. Emerging artificial visual sensory systems, utilizing in-sensor or near-sensor computing architecture, can incorporate both sensing and processing functions and simplify the system layout and fabrication. However, these systems still depend on silicon fabrication and packaging methods to create integrated sensory-computing architectures with interconnections. In terms of sensing functionalities, these visual systems can achieve various functions of visual perception, such as motion and movement perception (inspired by the fly's compound eye), panoramic and amphibious imaging (inspired by the crab's compound eye), wide field-of-view detection (inspired by the locust's compound eye), and scotopic/photopic adaptation (inspired by the retina). Certain bioinspired visual sensory systems implement visual perception functionalities at the device level using memristors or phototransistor arrays to enable sensory processing or sensory memory. Their potential applications are mostly limited to machine vision or computer vision, which involve tasks such as motion detection, movement recognition, image recognition, and color perception.

In contrast, our neuromorphic antennal sensory system has several key features that distinguish it from the recently reported artificial visual sensory systems.

(1) **Multimodal sensory input**: our system is inspired by the sensory organ of the insect's antennae, utilizing multimodal sensory input of both tactile and magnetic stimuli. Therefore, the sensory modality in our system is different from the visual modality in the visual systems mentioned above.

(2) **Biomimetic flexible 3D structure**: the sensor structure in our system employs a biomimetic, flexible, three-dimensional (3D) structure. This innovative structure is manufactured through low-cost, scalable fabrication techniques suitable for flexible electronics, allowing the entire sensor prepared in portable and wearable forms.

(3) **Diverse sensing functions**: the sensing functions of our system extend beyond visual perception, encompassing vibrotactile and magneto perception, spatiotemporal recognition, and sensory memory. Besides, our system adopts the receptor-neuron architecture and employs the fast adapting (FA) and slowly adapting (SA) encoding strategy of mechanoreceptors, imitating the neural pathway and neuronal coding of insect antennae.

(4) **Advanced sensory processing**: sensory processing of the sensor signal is achieved using ion-gated synaptic transistors, which are specialized for synaptic processing of tactile and magnetic stimuli with static or dynamic characteristics.

(5) **Wide range of applications**: our system enables a wide range of applications, including profile classification, Braille recognition, surface discrimination, material classification, magnetic navigation, and touchless interfacing. These applications are important for achieving tactile intelligence and perceptual augmentation in sensory robotics and smart interfaces.

In summary, our neuromorphic antennal sensory system stands apart from currently reported artificial visual sensory systems in the aspect of sensory modality, sensor structures, sensing functionalities, neuronal functions, sensory processing, and potential applications.

Supplementary Note 13
Limitations on active motion

Literature articles on the topic of antennal movement, antennal sensorimotor function, and active tactile exploration underscore the active and purposeful movement of insect antennae. Antennae share many similar morphological, neurobiological, and functional characteristics with the leg, since mechanosensory feedback is utilized in guiding the movement in both of them. The active movement capabilities and multimodal sensory functions of insect antennae play a pivotal role in various behaviors, including spatial orientation, search, tactile exploration, and communication. Depending on whether the size and structure of the antennae allow active sampling of external surfaces or objects through physical contacts, antennae can be categorized into contact antennae (e.g., in stick insects, ants, and honeybees) and non-contact antennae (e.g., in fly, mosquito, moth).

However, it is noteworthy that our work primarily focuses on the perception functions of the neuromorphic antennal sensory system rather than its motion control functions. The sensing experiments in our work were performed by passive movement to independently evaluate the tactile and magnetic sensation capabilities without using active exploration or active motion. This approach is justified by our system's intended applications in mobile robots or the human bodies, which inherently possess self-motion capabilities. Nevertheless, we recognize potential technical limitations in our current system regarding sensorimotor functions and active tactile exploration performance, and these limitations include: lack of adaptive control of the posture of the artificial antennae, unable to finely change the contact depth during tactile contact, and unable to achieve active touch or active contact. To address the limitations and develop the active motion capabilities, we propose a feasible strategy involving three key improvements to our current system:

(1) Geometry Optimization: The geometry of the electronic antennae sensor needs to be reduced and redesigned to meet the requirements of active tactile exploration tasks. Adjustments to aspect ratio and total length are essential.

(2) Integration of Flexible Actuator: A flexible actuator needs to be integrated into the artificial antennae to enable active movement. Soft or flexible materials with stimuli-responsive behavior, such as thermo actuating, electric actuating, or piezoelectric actuating, can be explored for this purpose.

(3) Closed-Loop Motion Control: Develop a closed-loop motion control strategy for the sensor array by drawing insights from the adaptive motor control of insect antennae. This aims to ensure reliable and efficient acquisition of mechanosensory information and guide the movement of the artificial antennae.

Supplementary Note 14

Preparation of the ion gel in the device

Preparation of the alginate ion gel started with dissolving sodium alginate (0.09 g) in an aqueous solution (2 wt.%, 6 ml) of acetic acid through vigorous stirring at an elevated temperature of 80 °C for one hour. Glycerol was added dropwise (6 droplets), and the mixture was allowed to cool down to room temperature. The obtained viscous, transparent mixture was ultra-sonicated for 10 min and then dropped onto the surface of a soda-lime glass slide. Heat treatment at 80 °C for one hour led to the formation of an ion-gel film, which was mechanically cut into desired sizes. The ion gel film was used to cover the channel and the planar gates of the artificial synaptic device, forming the dielectric layer, and an additional layer of transparent dressing could be laminated on top of the ion gel layer.

Supplementary Note 15

Fast-adapting (FA) and slowly-adapting (SA) spikes

In biology, fast-adapting (FA) and slowly-adapting (SA) spikes reveal different properties inherent to sensory input. Neuronal spikes characterized by FA and SA patterns are generated from different mechanoreceptors. Fast-adapting (or rapidly adapting) mechanoreceptor adapts rapidly to changes in stimuli (such as vibrations), producing transient responses. FA spikes are generated by fast-adapting mechanoreceptors during the start and end of dynamic stimulation. FA spikes exhibit "phasic" firing pattern characterized by rapid adaptation and prompt return to baseline pulse rates. Conversely, slowly-adapting (SA) mechanoreceptors produce sustained responses to static stimulation (such as pressure). SA spikes are generated by slowly-adapting mechanoreceptors throughout the entire duration when a static stimulus is presented. SA mechanoreceptors exhibit "tonic" firing patterns, characterized by a slow return to their normal firing rates. In terms of sensory functions, fast-adapting mechanoreceptors with FA spikes are useful in sensing textures or vibrations, while slowly-adapting mechanoreceptors with SA spikes play a pivotal role in perceiving position, movement, shape, and pressure.

Inspired by the sensory encoding mechanism observed in biological sensory systems, our work encodes sensory information into SA and FA spikes to capture static and dynamic characteristics of stimuli, respectively. SA spikes with tonic firing patterns are utilized for recognizing slow-changing sensory input with static characteristics, such as profile information and magnetic interactions. In contrast, FA spikes with phasic firing patterns are chosen for tasks involving rapid-changing sensory input with dynamic characteristics, such as vibration information. Consequently, the selection of SA or FA spikes is task-specific, ensuring reliable and accurate perception: SA spikes are employed for sensing tasks including chess profile classification and magneto-perception, where a slow response is essential for identifying profile information or interaction force. FA spikes are utilized for sensing tasks including surface pattern classification and material texture discrimination, where a fast response is necessary to identify the textures and patterns with high resolution. It is noteworthy that our system can simultaneously address SA and FA spikes, both encoded from the sensor signal and selectively processed in the synaptic devices. The design of our system based on the receptor-neuron model allows quantification of static and dynamic characteristics of spatiotemporal stimuli through synaptic currents recorded from the SA and FA devices. For accurate and reliable recognition of sensory signals, one type of sensory spike is chosen, leading to a biased sensory processing. This strategy is analogous to the perceptual weighing function observed in the brain, wherein the biased assignment of perceptual weights to specific sensory stimuli depends on the reliability and intensity of sensory inputs.

In general, slow-adapting (SA) and fast-adapting (FA) sensory spikes correspond to the static and dynamic properties of the sensory input. Both SA and FA spikes are sensory spikes carrying the spatiotemporal information of the sensory stimuli. At system level, our neuromorphic system can simultaneously process two types of sensory spikes. For a specific perception task, the appropriate sensory spike is manually selected based on the changing behavior of the sensory stimuli, in order to improve the reliability and accuracy of the perceptual recognition.

For further improvement, we propose a feasible method for the dynamic adjustment of the spike selection strategy. This involves evaluating the mean firing rates of sensory spikes during a tactile perception event. Specifically, the microcontroller in the spike-encoding circuit can be programmed to compare the mean firing rates of FA and SA spikes. By incorporating this function into the microcontroller, the neuromorphic system can achieve the dynamic adjustment of the spike selection strategy. This dynamic adjustment is performed in an event-based manner to select reliable sensory spike based on mean firing rate, and it can potentially address the changes in the environments or task requirements.

As an extension, to realize the learning capabilities in terms of adjusting the spike selection strategy, we propose an approach inspired by the multilayer perceptron (MLP) model with backpropagation functions. This approach involves the backward transmission of information from a later layer to a previous layer, thus reducing recognition error and enabling the learning function. To achieve the perception learning function, our system can be further integrated with a feedback circuit that forwards the device output to the microcontroller in the spike-encoding circuit. This integration forms feedback loops and allows the bi-directional flow of sensory information, similar to the notion of recurrent neural network (RNN) in machine learning. The microcontroller can be programmed to evaluate the outputs from the SA and FA devices, and then select the suitable sensory spike for sensory processing and recognition.

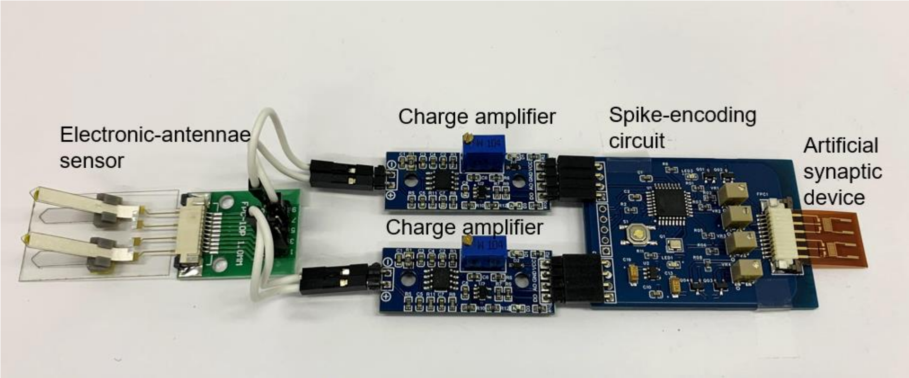

Figure S1. Photograph of the electronic antennal nerve system. The system includes an electronic antennae sensor, charge amplifiers, a spike-encoding circuit, and artificial synaptic devices.

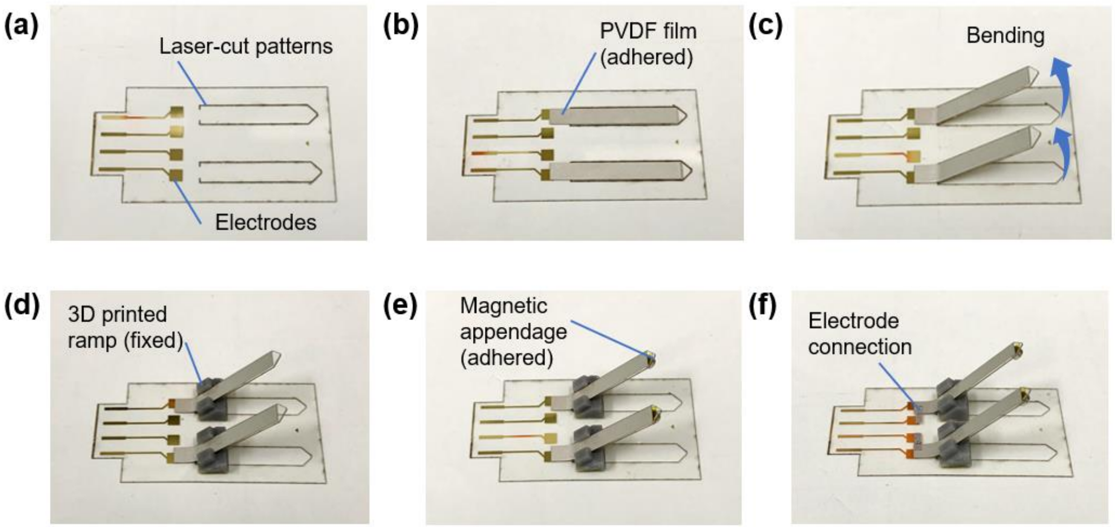

Figure S2. Fabrication process of the electronic-antennae sensor. (a) Laser cutting of PET substrate followed by electrode fabrication. (b) Lamination of PVDF piezoelectric film using double-sided adhesive tape. (c) Out-of-plane bending of the antennae patterns. (d) Installation of the plastic ramps. (e) Adhering of the magnetic appendages to the tip of the artificial antennae. (f) Connecting the piezoelectric films to the electrodes using conductive tape and conductive paste. The plastic ramps were fabricated by stereolithography 3D printing technique using black or gray resin. Two disc-shaped strong magnets that are axially magnetized were used as the magnetic appendages, and their magnetization directions were kept in opposite directions during installation. The PET substrate (XF-162) was purchased from XFnano Materials, and the PVDF films (52 µm with metalized surface) were purchased from TE Connectivity.

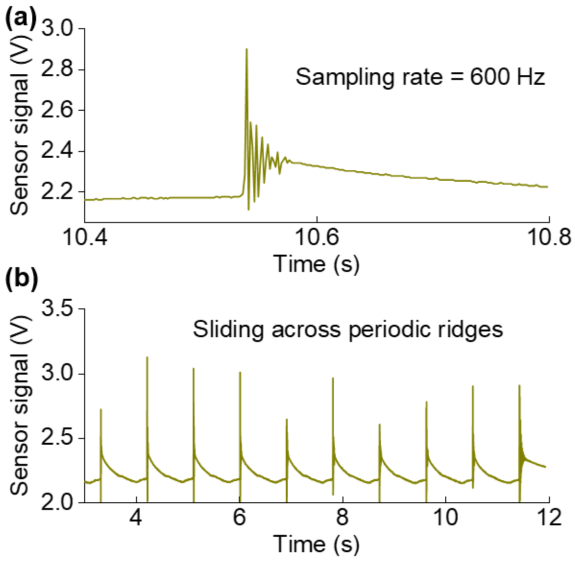

Figure S3. Lateral-sliding induced sensor signal recorded at a high sampling frequency of 600 Hz. (a) Time-resolved sensor signal in response to lateral sliding across a single ridge. The high sampling rate at 600 Hz can finely reveal the oscillation nature of the sensor signal caused by the vibration of the flexible antennae structure in the sensor. (b) Time-resolved sensor signal in response to lateral sliding across periodic ridges.

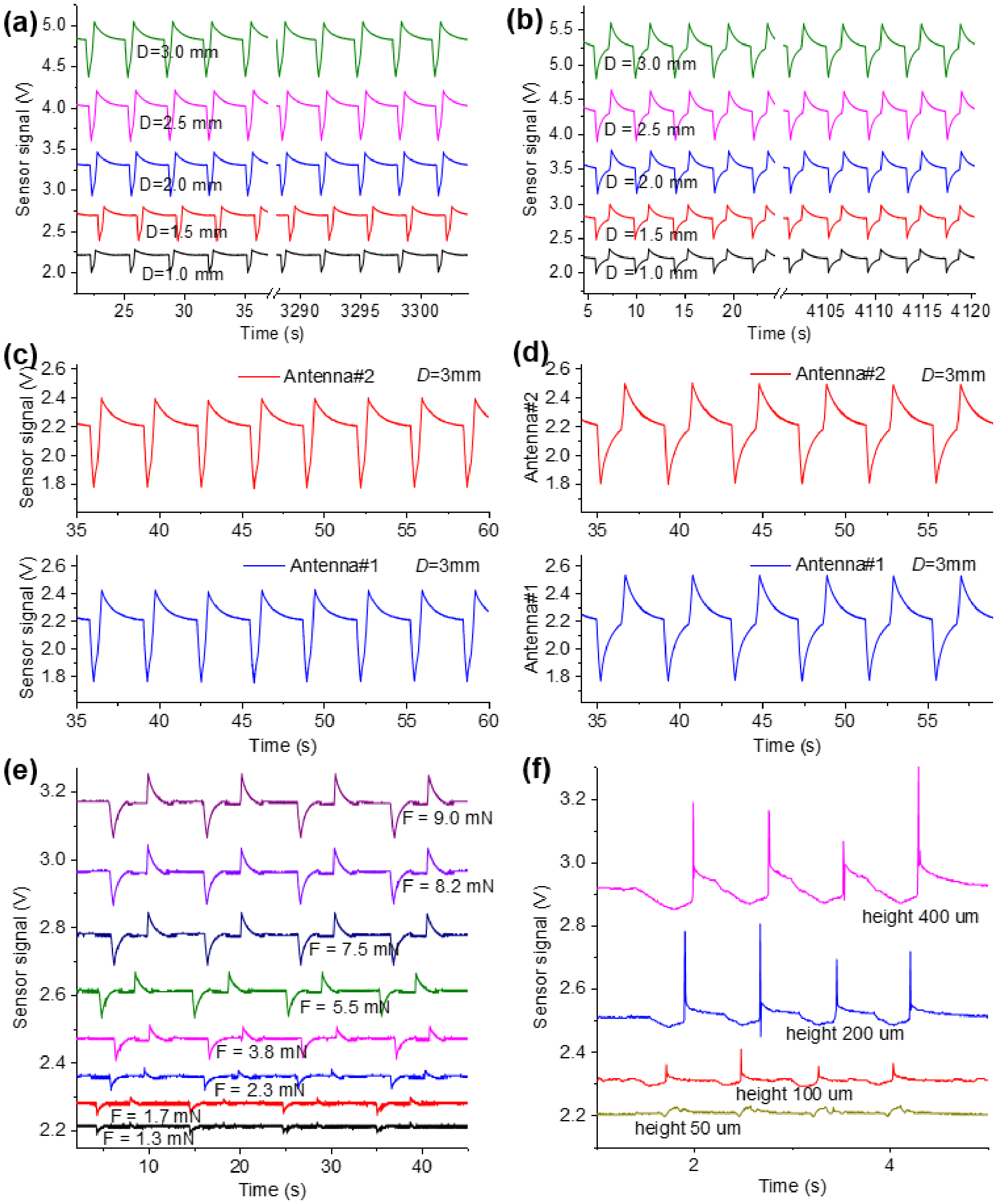

Figure S4. Mechano-sensation performance of the electronic-antennae sensor. (a–b) Operational stability of the sensor in response to repeated cycles (>1000) of tactile contact with no dwell time (a) and with a dwell time of 1 s (b). The experimental condition was changed by altering the deflection of the artificial antennae (from 1 mm to 3 mm). (c–d) Sensor-to-sensor variation evaluated by measuring the output from a pair of artificial antennae (antenna #1 and #2) under repeated cycles of tactile contact with no dwell time (c) and with a dwell time of 1s (d). (e) Time-resolved sensor signal in response to various applied forces (minimum value 1.3 mN) during tactile contact. (f) Time-resolved sensor signal in response to different surface heights (minimum value 50 µm) during vibrotactile sliding. The results above show high operational stability (> 1000 cycles), low sensor-to-sensor variation (< 2.5%), and low detection limit of the sensor (tactile force 1.3 mN; surface height

50 μm). The deflection of the artificial antennae is denoted by *D*, and the applied tactile force is represented by *F*. All the curves are offset for clarity.

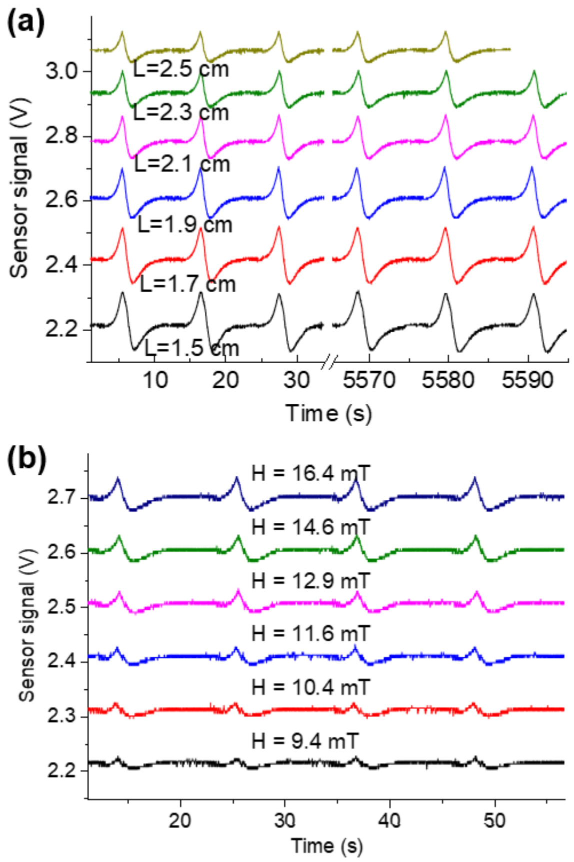

Figure S5. Magneto-sensation performance of the electronic-antennae sensor. (a) Operational stability (>500 times) of the sensor in response to non-contact magnetic stimuli (generated from a cylindrical magnet) with no dwell time. The experimental condition was changed by altering the sensor-to-object distance (from 1.5 cm to 2.5 cm). (b) Time-resolved sensor signal in response to various magnetic fields under non-contact mode with no dwell time. The minimum detection limit is 9.4 mT. *L* denotes the sensor-to-object distance (minimum value), and the applied magnetic field (peak value) is represented by *H*. All the curves are offset for clarity.

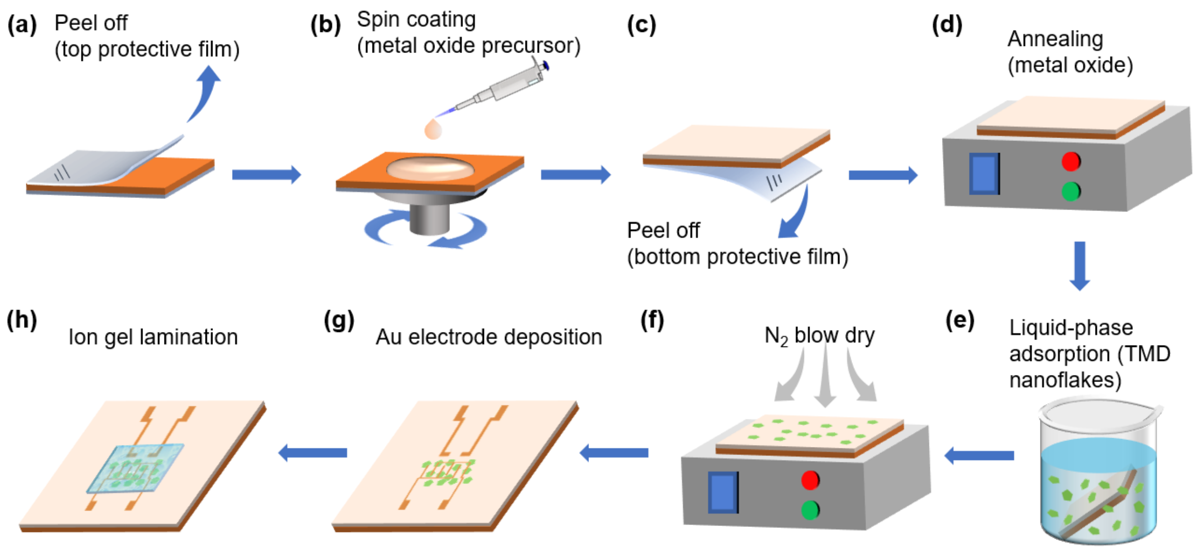

Figure S6. Fabrication process of the artificial synaptic device. (a) Peeling off the top protective film on the polyimide substrate. (b) Spin coating of metal oxide precursor on the top surface of a polyimide substrate. (c) Peeling off the bottom protective film on the polyimide substrate. (d) Annealing of the sample at elevated temperature. (e) Liquid-phase adsorption of TMD nanoflakes on the sample surface. (f) Blow dry using nitrogen gas. (g) Deposition of gold electrodes. (h) Lamination of ion gel on the sample surface.

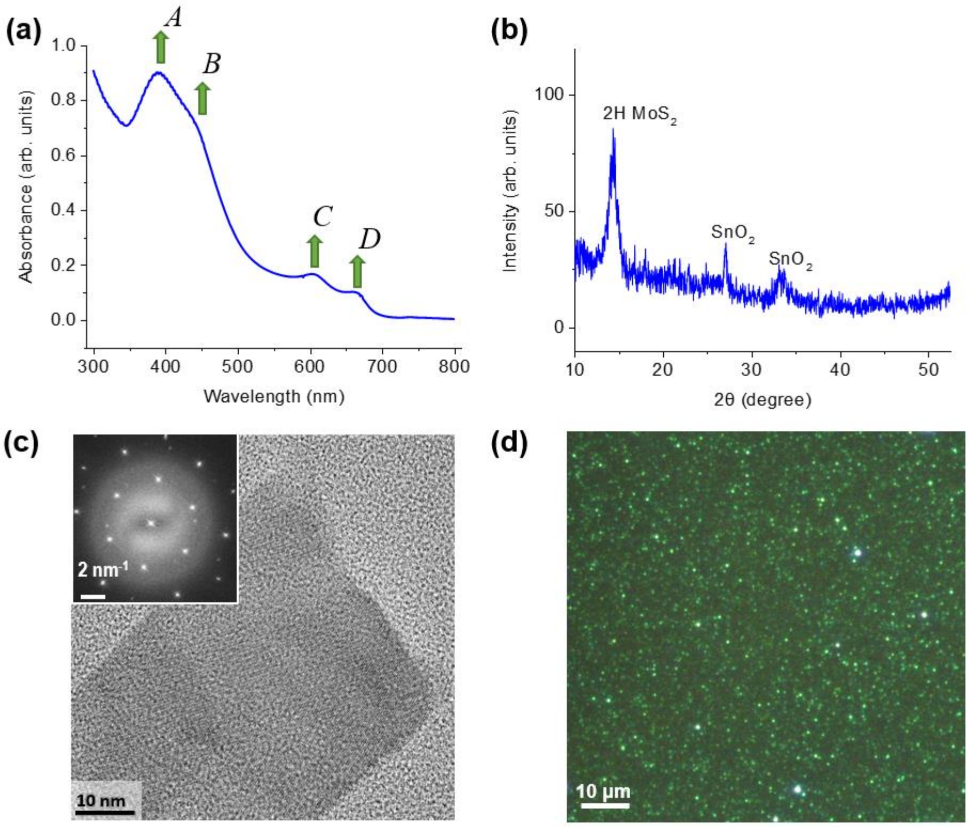

Figure S7. Characterization of the semiconductor materials in the artificial synaptic device. (a) UV–visible absorption spectra of the TMD nanoflakes dispersed in ethanol/water mixture. The peaks at 393, 448, 609, and 664 nm are the characteristic absorption bands of exfoliated few-layer MoS_2 (2H phase). (b) XRD patterns of the semiconductor material composed of metal oxide thin film and adsorbed nanoflakes. The result indicates the presence of SnO_2 and MoS_2. (c) TEM image and selected area electron diffraction (SAED) pattern of a TMD nanoflake. Crystalline structures of the MoS_2 nanoflake are confirmed by the SAED pattern in the inset (scale bar 2 nm^{-1}). (d) Dark-field optical microscopy image of the semiconductor channel revealing that the nanoflakes are distributed on the surface of the metal oxide film.

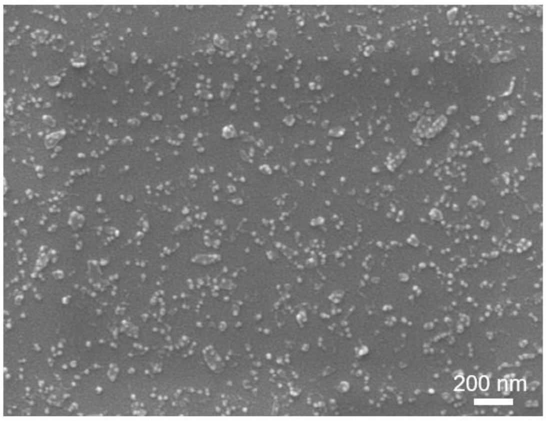

Figure S8. SEM image of the semiconductor channel in the artificial synaptic device. It is observed that nanoflakes sized at tens of nanometers are distributed on the smooth surface of the metal oxide thin film.

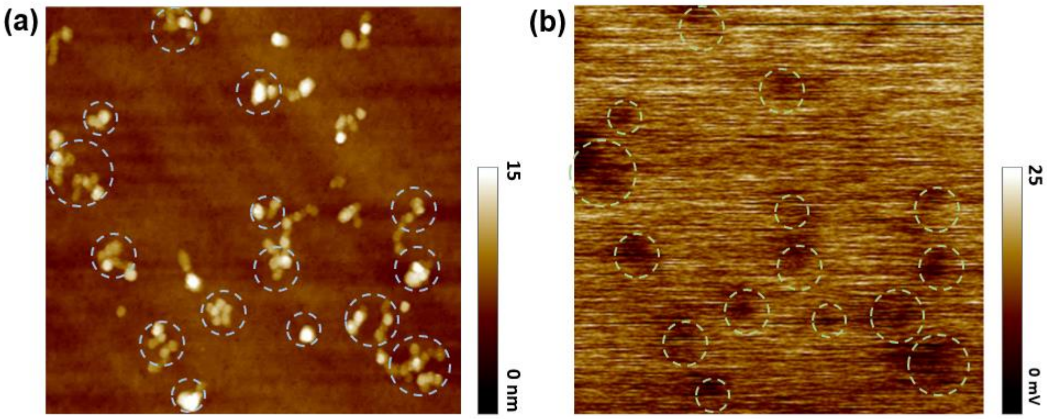

Figure S9. Surface potential of the semiconductor channel in the artificial synaptic device. (a) Topology image acquired by AFM. (b) Surface potential mapping obtained by Kelvin-probe force microscopy (KPFM). The regions of potential drop in (b) match well with the sites of the nanoflakes in (a), which are marked by dashed circles for comparison. The locations of the nanoflakes with potential change may promote change trapping.

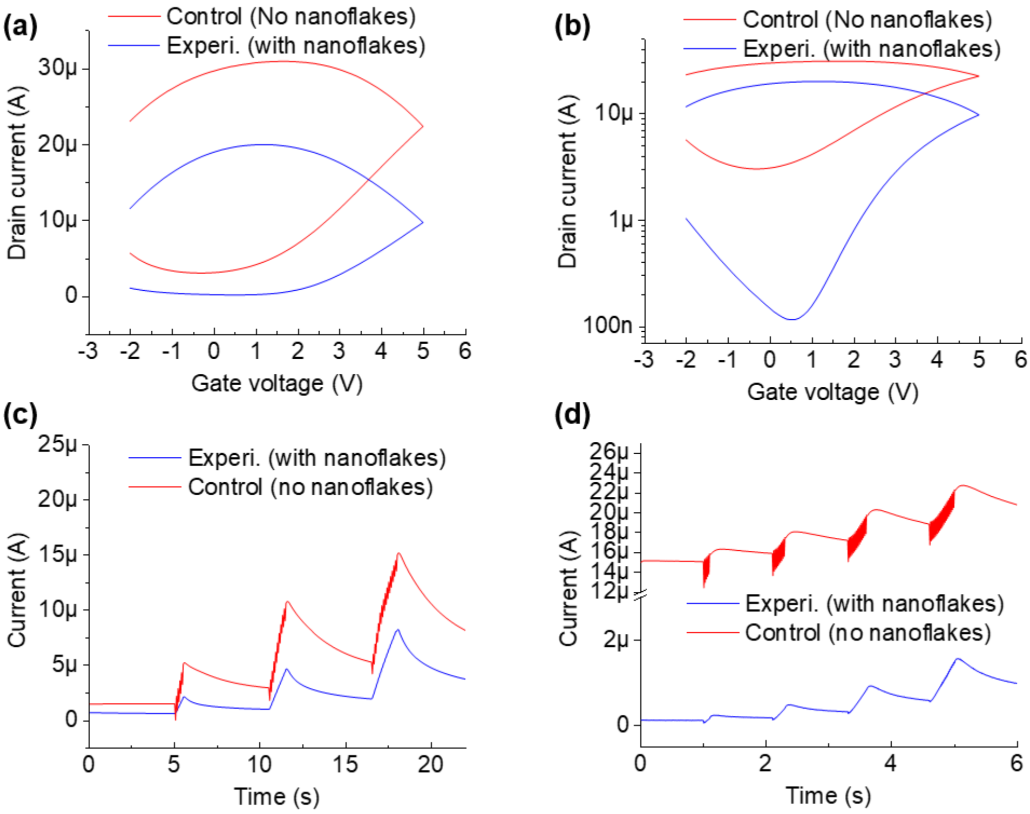

Figure S10. Performance comparison of the artificial synaptic device without and with the introduction of TMD nanoflakes. (a–b) Transfer curves of the two devices in linear scale (a) and log scale (b). (c–d) Spike-number dependent plasticity of the two devices operated under voltage spikes in 20-Hz low frequency (c) and 100-Hz high frequency (d). The devices were tested under a small bias voltage of 10 mV. By comparison, it can be observed that the introduction of TMD nanoflakes in the device increases the on-off ratio (from 7.5 to 84.5), suppresses the off-state current (from 3.05 to 0.15 μA), increases the linearity of potentiation behavior, and improves the signal to noise ratio of synaptic response. Therefore, liquid-phase adsorption of the TMD nanoflakes enhances the device's transistor and synaptic characteristics, which are favorable for ionic gating and conductance modulation. Besides, shifts in the threshold voltage (from 1.0 to 2.4 V) were observed in the transfer curve, and it can be related to the built-in field arising from carrier trapping.[4]

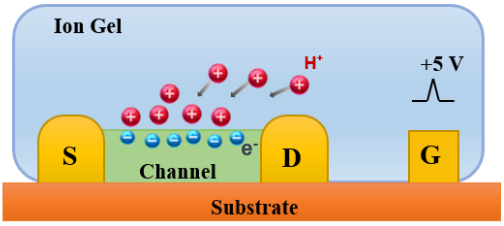

Figure S11. Illustration of the synaptic potentiation process of the device. This schematic illustration shows the transistor-type artificial synaptic device with a strong lateral protonic/electronic coupling effect under the stimuli of voltage spikes. The planar gate (applied with positive spikes) is regarded as the presynaptic input terminal of a synapse; the source/drain terminals are regarded as the postsynaptic output terminals of the synapse; the semiconductor channel (N-type) is analogous to the synaptic cleft transmitting neurotransmitters. The conductance modulation process in the artificial synaptic device mimicked the synaptic potentiation behavior.

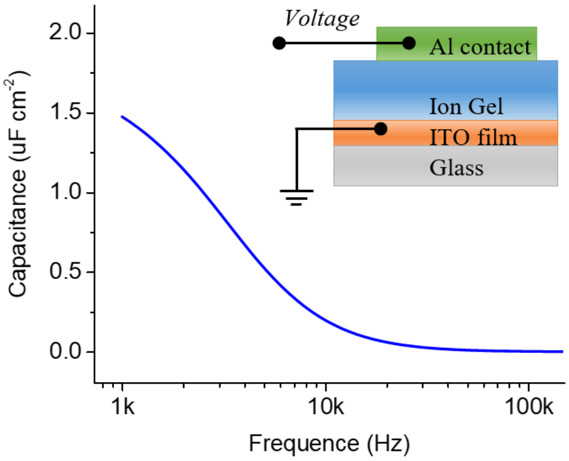

Figure S12. Frequency-dependent specific capacitance of the ion gel film. The inset shows the *C-f* measurement setup, where the ion gel film was sandwiched between ITO glass and Al contact. The thick ion gel film exhibits a large specific capacitance of ~1.5 μF cm^{-2} at a frequency of 1 kHz, and this large capacitance derives from the formation of electrical double layers at interfaces. The capacitance associated with the ion gel film is also strongly dependent on frequency up to 10 kHz since the mobilities of ions limit the polarization response time. Double-layer capacitances for liquid electrolytes typically have formation times of the order of 10 μs. The specific capacitance (~1.5 μF cm^{-2} at 1 kHz) is comparable to ceramic gate insulators (HfO_2).[5]

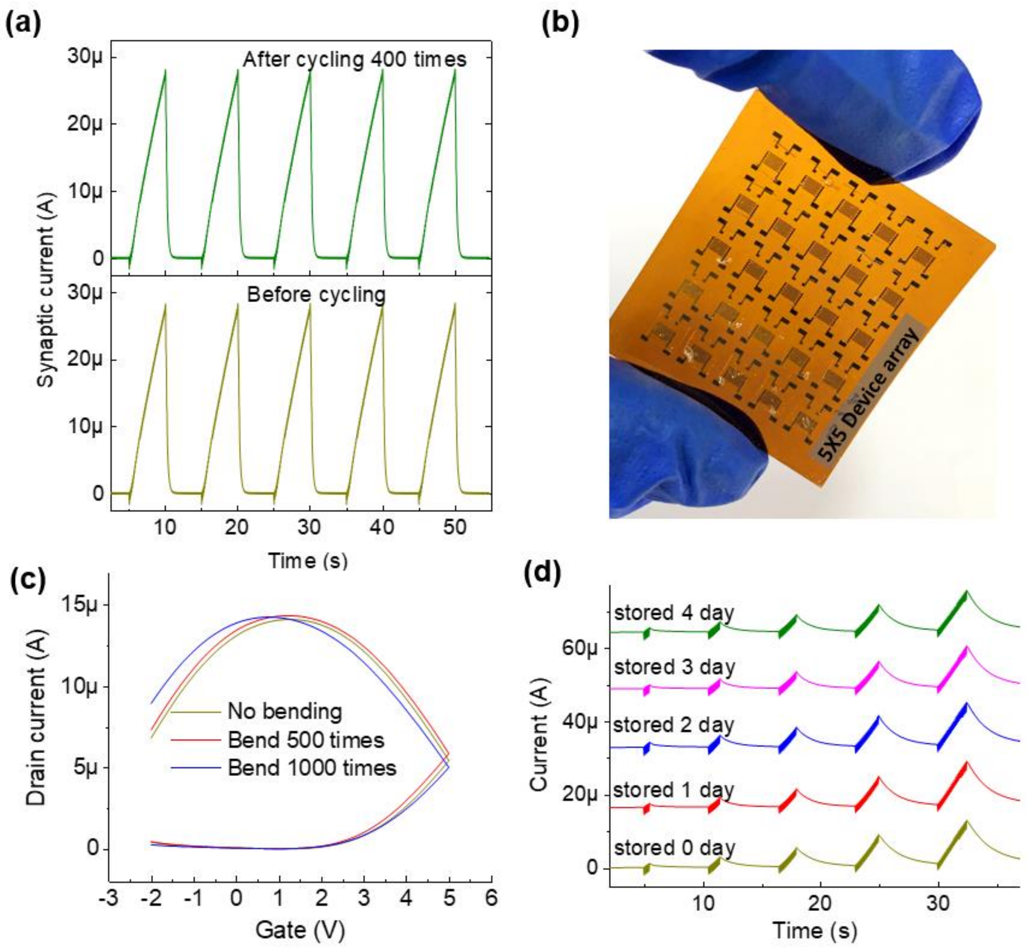

Figure S13. Cycling stability, operational stability, and bending stability of the artificial synaptic device. (a) Cycle-to-cycle variation of the device evaluated by repeatedly applying 50 positive and 50 negative spike trains at 10 Hz for over 400 cycles. The synaptic current shows small variation (<2%). (b) Photograph of the device array fabricated in a 5 × 5 matrix. (c) Transfer curve of the device measured after repeated bending for 0, 500, and 1000 times with a bending radius of 2 cm. (d) Synaptic characteristics (spike-number dependent plasticity) of the device measured after storage for 0, 1, 2, 3, and 4 days. The devices were tested under a small bias voltage of 10 mV. The curves in (d) are offset for clarity.

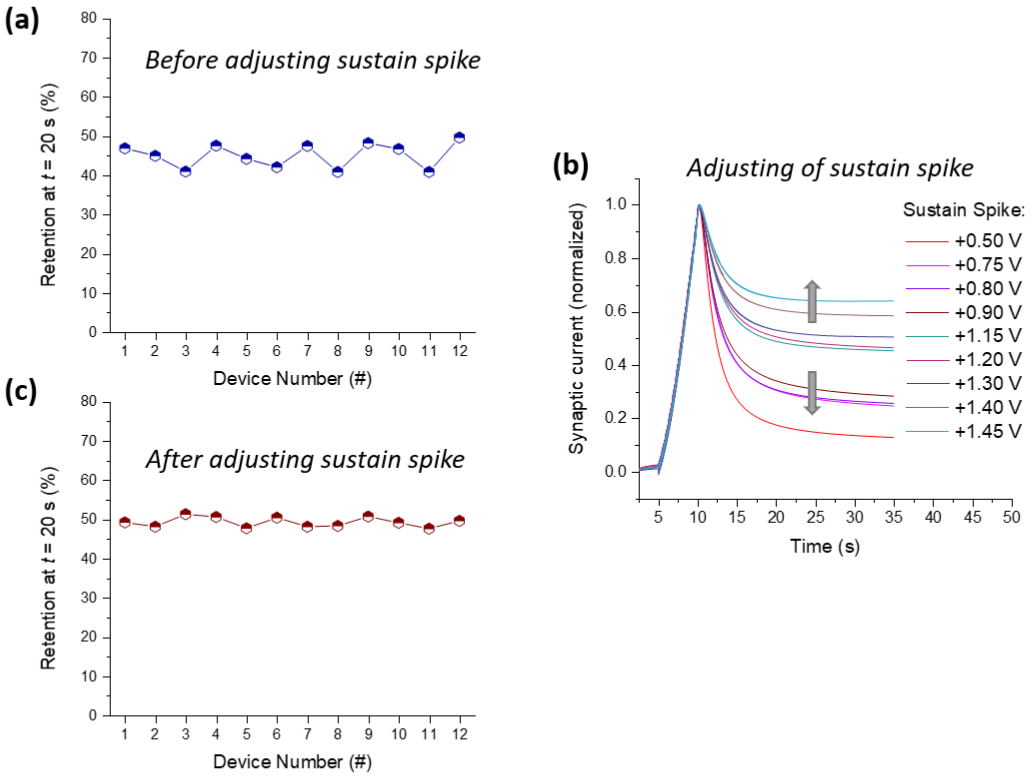

Figure S14. Device-to-device variations of the artificial synaptic device. (a) Device-to-device variation of 12 devices evaluated by measuring their memory retention behaviors ($t = 20$ s) before implementing the strategy of adjusting sustaining spikes (+5 V sensory spikes followed by sustaining spikes with fixed amplitude of +1 V). (b) Implementation of the strategy of adjusting sustaining spikes showing that the memory retention behavior of the device can be finely regulated. (c) Device-to-device variation of 12 devices evaluated by measuring their memory retention behaviors ($t = 20$ s) after implementing the strategy of adjusting sustaining spikes (+5 V sensory spikes followed by sustaining spikes with varied amplitude).

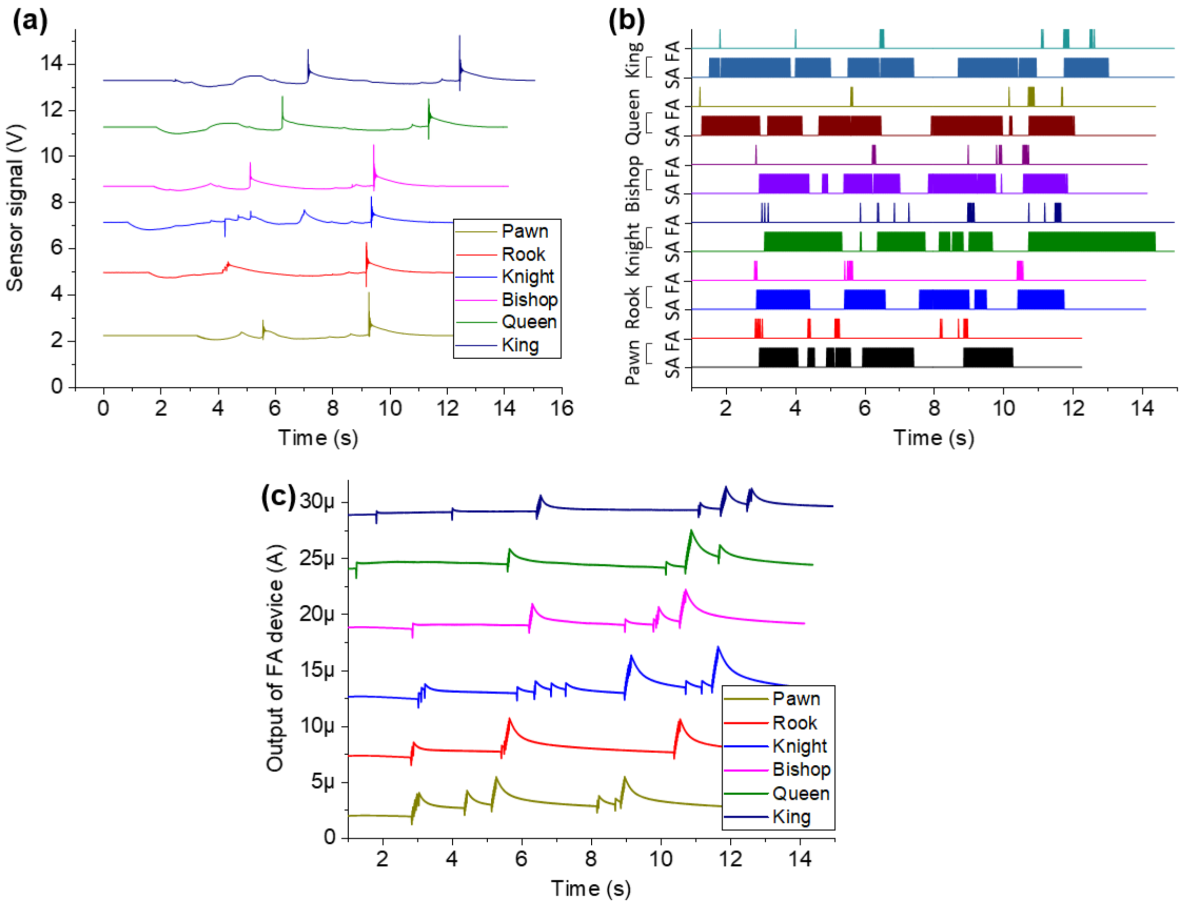

Figure S15. Sensor signals, encoded spikes, and device outputs for chess profile classification. (a) Time-resolved sensor signal. (b) Patterns of the SA and FA spike trains. (c) Time-resolved output of the FA device. Six different types of chess pieces, including pawn, rook, knight, bishop, queen, and king, were detected using a single artificial antenna aligned to each chess piece's axial center. All the curves are offset for clarity.

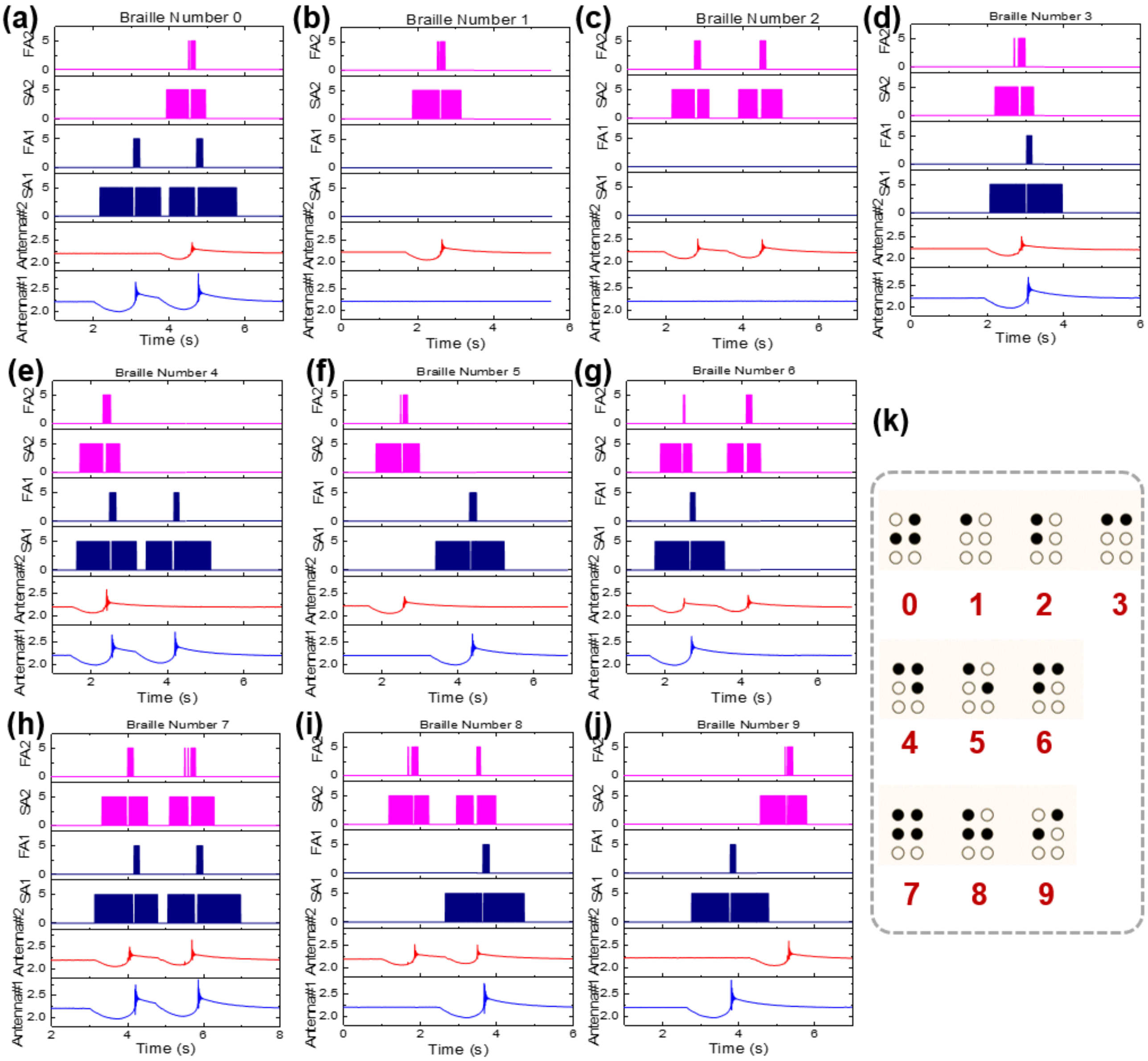

Figure S16. Sensor signals and encoded spikes for Braille code detection. (a–j) Time-resolved sensor signals and the corresponding SA and FA spike trains from two artificial antennae (antenna #1 and #2) when Braille code of “0” (a), “1” (b), “2” (c), “3” (d), “4” (e), “5” (f), “6” (g), “7” (h), “8” (i), and “9” (j) was detected, respectively. SA1 and FA1 spikes were encoded from antenna #1; SA2 and FA2 spikes were generated from antenna #2; SA1 and SA2 spikes were transmitted to the SA device; FA1 and FA2 spikes were sent to the FA device. The ten different Braille codes were detected using a pair of artificial antennae, and each artificial antenna was in contact with one column of Braille dots. The pitch of the dot columns (8 mm) was slightly smaller than that of the artificial antennae (10 mm). Consequently, the sensor signals from the two artificial antennae exhibited temporal incongruence, which is beneficial for classifying symmetric patterns. (k) Braille codes corresponding to ten numbers (“0” to “9”). The number and arrangement of the six dots in a 3 × 2 matrix distinguishes one character from another.

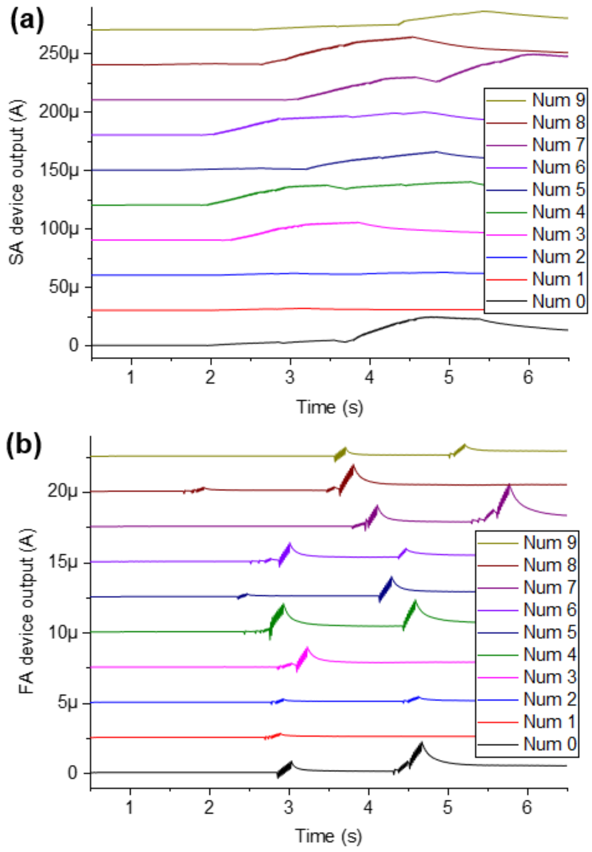

Figure S17. Output of the artificial synaptic device for Braille code classification. (a) Time-resolved output of the SA device. (b) Time-resolved output of the FA device. Ten different Braille codes (from "0" to "9") were detected using a pair of artificial antennae, and each artificial antenna was in contact with one column of Braille dots. All the curves are offset for clarity.

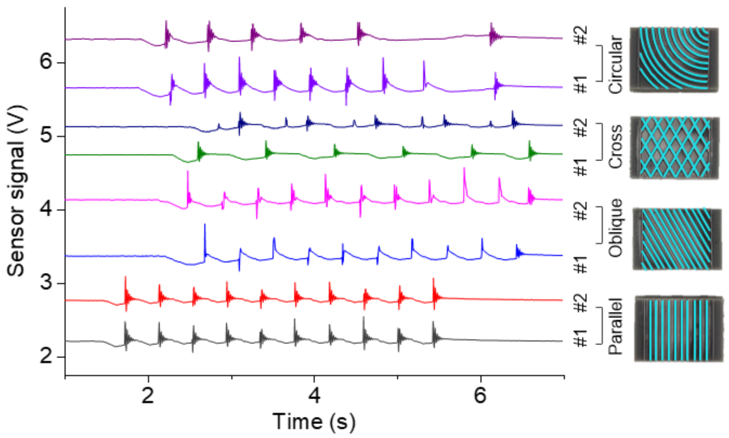

Figure S18. Sensor signals for the classification of customized ridge patterns. The plot shows the time-resolved sensor signals from two artificial antennae (antenna #1 and #2) when parallel, oblique, crisscross, and circular patterns (guided by light blue lines) were detected. All the curves are offset for clarity.

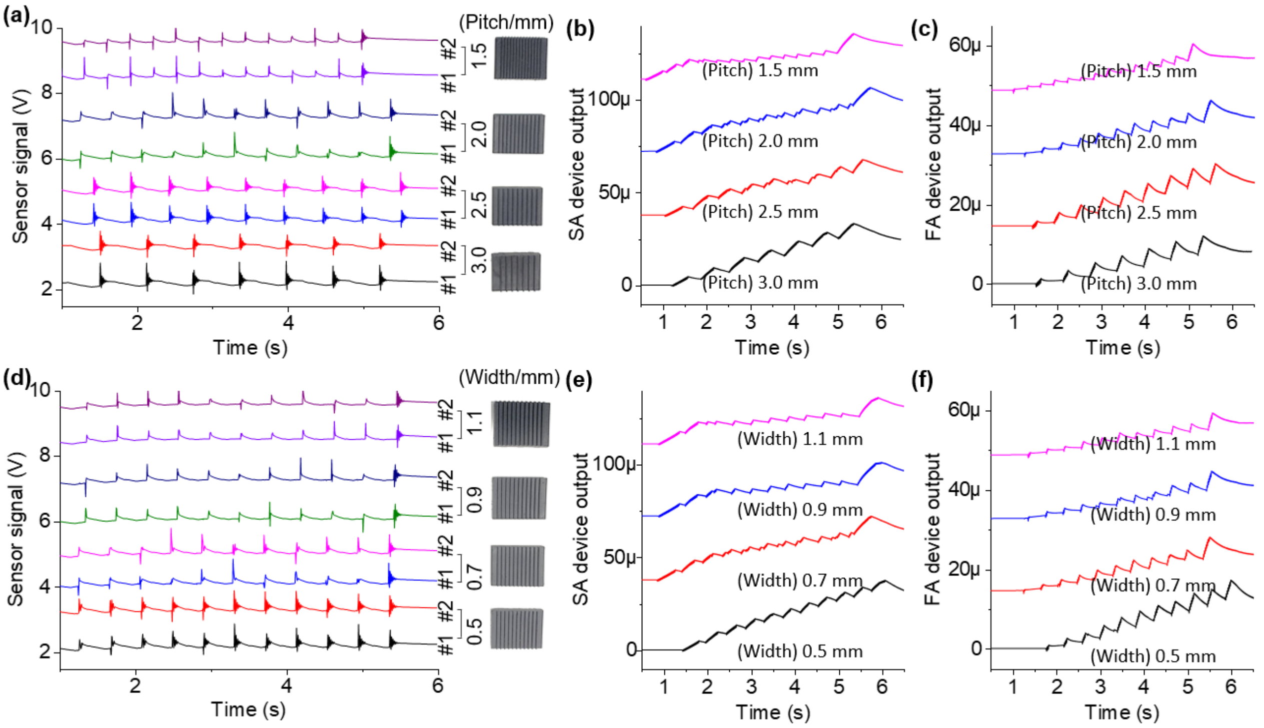

Figure S19. Sensor signals and device outputs for surface pattern classification when the pitch or width of the ridge pattern was changed. (a–c) Sensor signals (a), SA device output (b), and FA device output (c) acquired by changing the pitch of parallel ridges from 3.0 mm to 1.5 mm. (d–f) Sensor signals (d), SA device output (e), and FA device output (f) obtained by changing the width of parallel ridges from 0.5 mm to 1.1 mm. All the curves are offset for clarity.

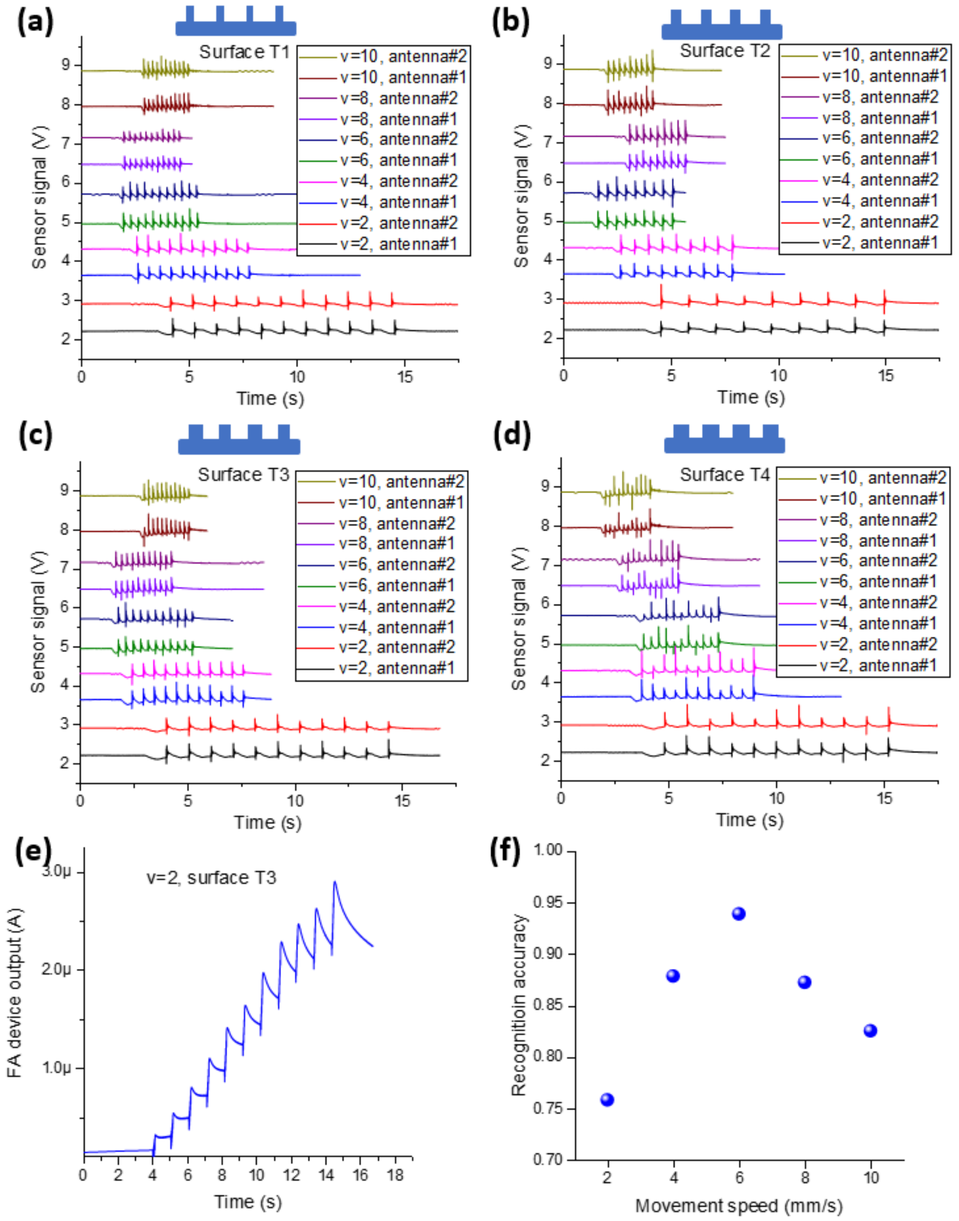

Figure S20. Influence of the scanning speed of the sensor on surface recognition performance. (a–d) Sensor signals acquired at various scanning speed of the sensor when surface patterns with ridge widths of 0.5 mm (a), 0.7 mm (b), 0.9 mm (c), and 1.1 mm (d) were laterally scanned. (e) Representative output of the FA device showing the stepwise spiking, corresponding to the periodic stripes of the sample surface. (f) Relationship between the scanning speed of the sensor and the accuracy of surface pattern recognition.

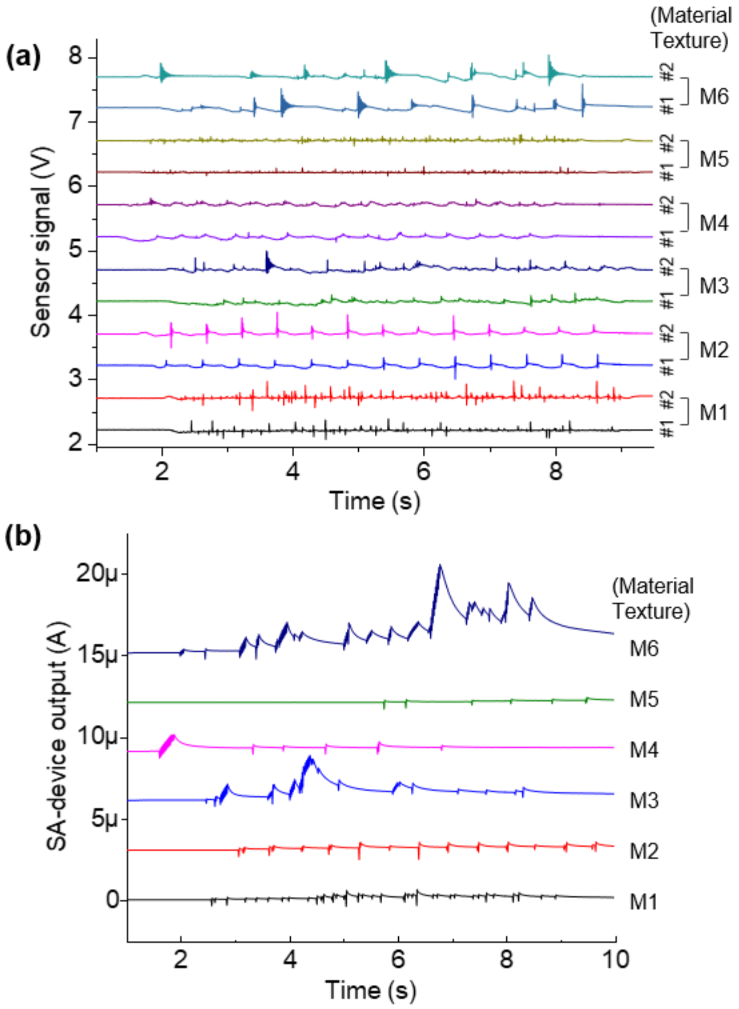

Figure S21. Sensor signals and device outputs for material texture classification. (a) Time-resolved sensor signal from two artificial antennae (antenna #1 and #2). (b) Time-resolved output of the SA device. Six different material textures (M1: metal foam, M2: patterned plastic, M3: dish sponge, M4: canvas fabric, M5: abrasive paper, M6: porous sponge) were detected using a pair of artificial antennae. All the curves are offset for clarity.

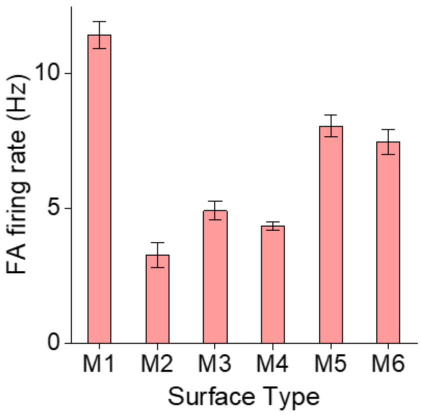

Fig. S22 Mean firing rate of FA spikes for material texture classification. Six different material textures (M1: metal foam, M2: patterned plastic, M3: dish sponge, M4: canvas fabric, M5: abrasive paper, M6: porous sponge) were detected using a pair of artificial antennae. The experiments were performed for multiple trials, and the error bars in this figure represent the standard deviation.

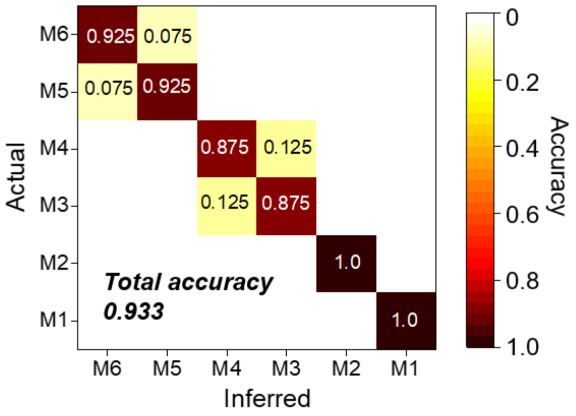

Fig. S23 Results of material texture classification shown as a confusion matrix. The ending value of the FA device's synaptic current was used as the classification criteria, and the classification process adopted a decision-tree strategy, including training and inferring procedures. Six different material textures (M1: metal foam, M2: patterned plastic, M3: dish sponge, M4: canvas fabric, M5: abrasive paper, M6: porous sponge) were tested using a pair of artificial antennae. The total accuracy of the material texture classification using our system is 93.3%, even higher than that of human participants (average accuracy of 88.2%) who performed "blind" tactile exploration without visual cues.

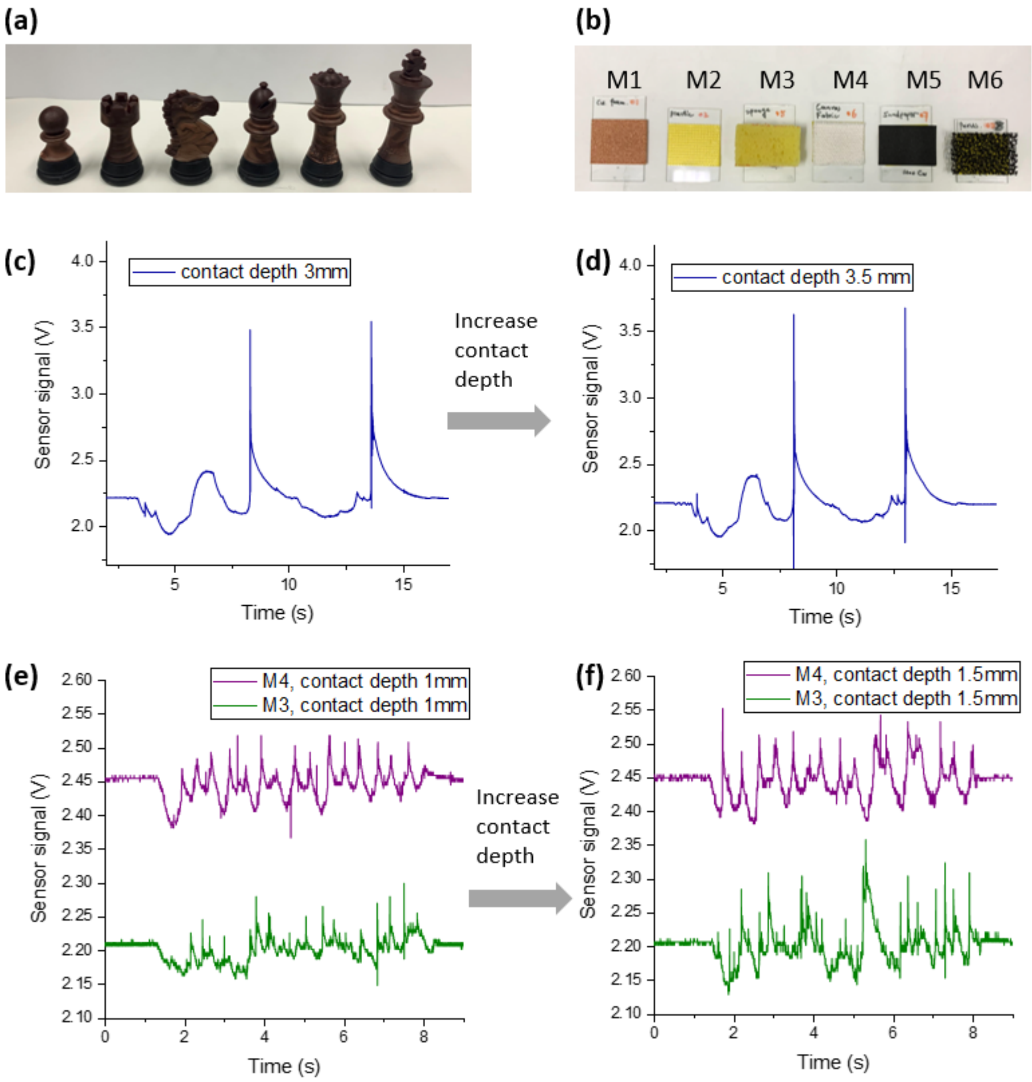

Figure S24. Further improvement of tactile recognition accuracy. (a) Photograph of the six chesses used for profile classification. (b) Photograph of the six material textures (M1: metal foam, M2: patterned plastic, M3: dish sponge, M4: canvas fabric, M5: abrasive paper, M6: porous sponge) used for surface texture discrimination. (c–d) Sensor signal acquired by scanning the chess profile of "King" at contact depth of 3 mm (c) and 3.5 mm (d). (e–f) Sensor signal acquired by scanning the surface texture of M3 and M4 at contact depth of 1 mm (e) and 1.5 mm (f). The experimental results confirm that appropriately increasing the contact depth may improve the quality of the sensor signal, which may presumably improve the recognition accuracy of perception tasks.

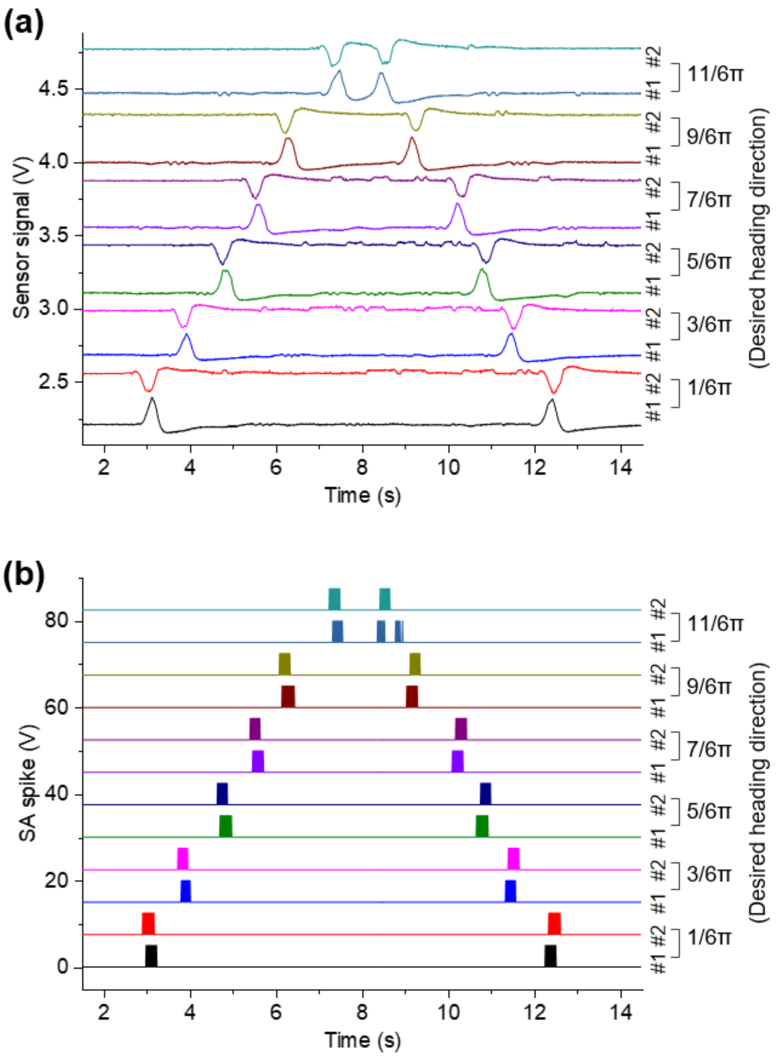

Figure S25. Sensor signals and encoded spikes for robot navigation tasks. (a) Time-resolved sensor signal from two artificial antennae (antenna #1 and #2). (b) Spatiotemporal patterns of the encoded SA spikes. Different desired heading directions (1/6π, 3/6π, 5/6π, 7/6π, 9/6π, 11/6π) were tested using a pair of artificial antennae, which was installed upside down on the front arm of a mobile robot. All the curves are offset for clarity.

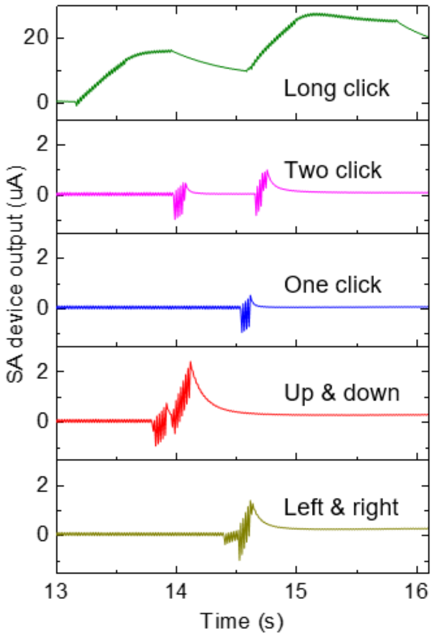

Figure S26. Output of the SA device for finger-motion classification tasks. The plot shows the time-resolved synaptic currents from the SA device when finger motions of long click, double click, single click, up/down, and left/right were detected.

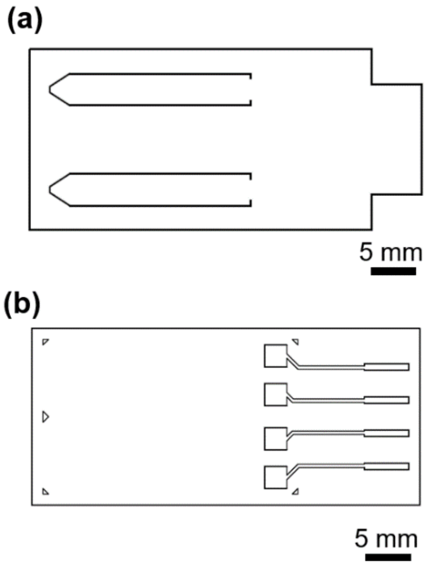

Figure S27. Antennae pattern used for sensor fabrication. (a) Laser-cutting pattern of the artificial antennae. (b) Electrode pattern of the electronic-antennae sensor.

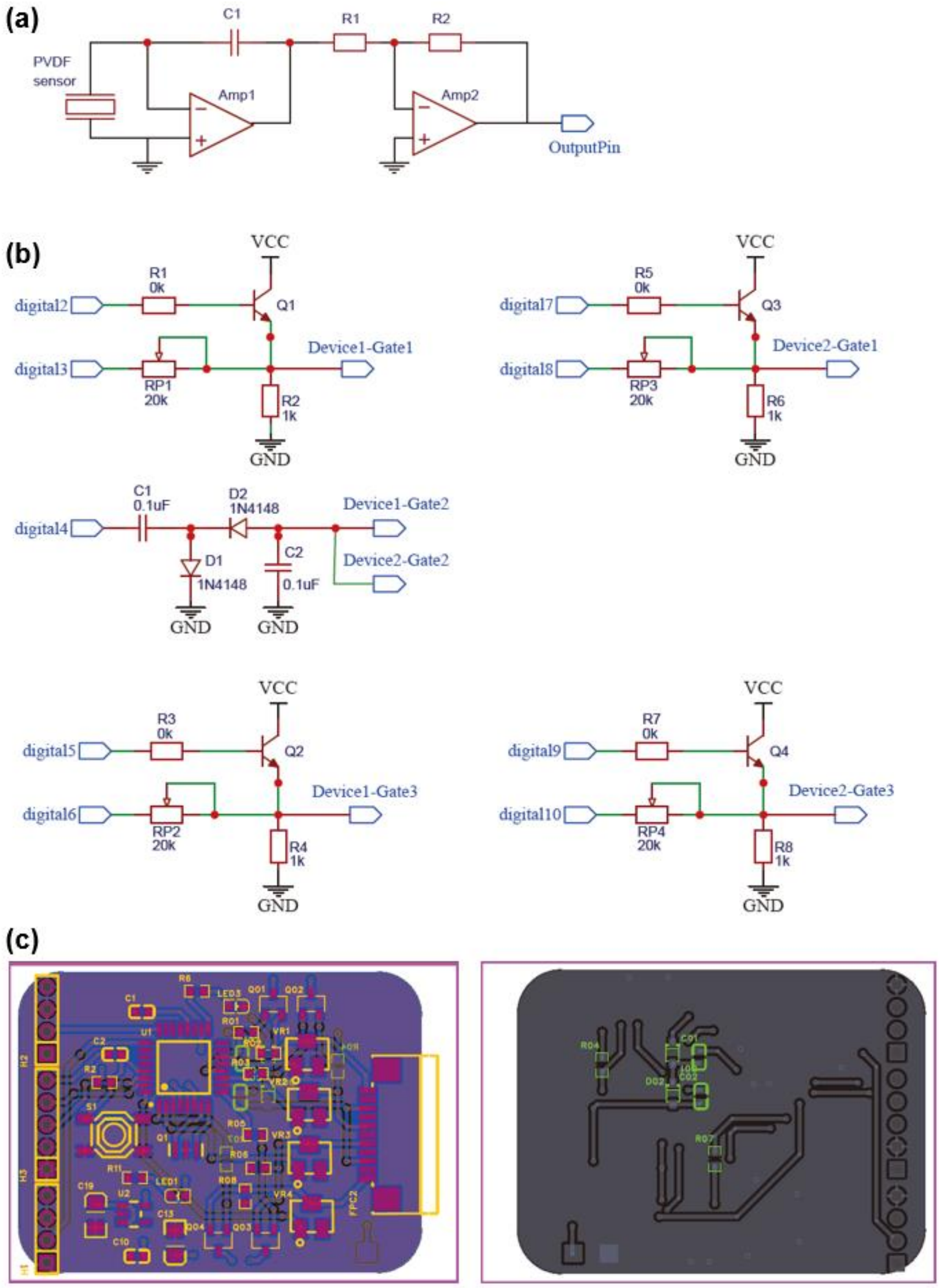

Figure S28. Diagram of the peripheral circuit in the electronic antennal nerve system. (a) Diagram of the charge-to-voltage converter (charge amplifier), which converts the sensor's piezoelectric signal to voltage signal. (b) Diagram of the spike-encoding circuit, which receives signals from the microcontroller through digital pins and sends sensory spikes (positive) or reset spikes (negative) to two artificial synaptic devices. (c) PCB layout of the spike-encoding circuit containing a microcontroller chip.

Table S1. Comparison of recently reported synaptic devices (array) fabricated using solution-processable materials, including precursors, organics, and nanomaterials. BCM: Bienenstock–Cooper–Munro learning rule; LIF: leaky integrate and fire; TMD: transition metal dichalcogenide.

**Type of device**	**Materials**	**Fabrication methods**	**Synaptic characteristics**	**Neuronal functions**
Artificial neuron of memristive device array [6]	Metal oxide (TiO_x) nanosheets	Dip-coating assembly	Resistive switching	LIF behavior
Flexible optoelectronic memristive synapses [7]	Black phosphorus flakes	Drop-casting	Spike-dependent plasticity, memory storage	Visual perception, visual memory
Hetero-structure memristive device [8]	2D nanoflake (MoO_x/MoS_2 and WO_x/WS_2)	Wet coating (LB), oxidation	Bipolar resistive switching, memory retention	—
Fully printed optoelectronic synaptic transistors [9]	Metal oxide precursors, quantum dots (InP/ZnSe)	Inkjet and screen printing	Spike-dependent plasticity, memory retention	Visual processing, visual recognition (simulated)
One-dimensional organic artificial multi-synapses [10]	Organics (PVDF-TrFE and pentacene)	Dip-coating (physical deposition required)	Spike-dependent plasticity, memory retention	Pattern recognition of handwriting and heartbeat (simulated)
Two-terminal threshold switching memristor [11]	Quantum dots ($CsPbBr_3$), black phosphorus nanosheets	Material self-assembly, spin-coating	Threshold switching	Visual processing, perception
Flexible carbon nanotube synaptic transistor [12]	Carbon nanotubes	Liquid-phase functionalization (physical deposition required for dielectric)	Spike-dependent plasticity, memory	Mechano-sensation
Biaxially stretchable elastomeric synaptic transistors [13]	Organics (P3HT, SEBS)	Drop-casting, spin-casting	Spike-dependent plasticity, memory retention	Neuromorphic processing of strain and visual input
Organic synaptic transistor [14]	Organics (conjugated polymer)	Drop-casting, spin-coating	Spike-dependent plasticity, multi-integration	Mechano-sensation
Flexible multi-gate artificial synaptic transistor array (this work)	Metal oxide precursor, small TMD nanoflakes (MoS_2)	Spin coating, liquid-phase adsorption	Spike-dependent plasticity, BCM, adjustable memory, multi-integration	Spatiotemporal processing; mechano- and magneto-perception

Table S2. Chess profile classification results obtained from human participants. The human subjects (N = 6) performed "blind" tactile exploration without visual cues during the experiments. Each participant performed multiple trials (55 trials) for different types of chess pieces. The average value and standard deviation are 87.3% and 1.6%, respectively.

Participant	Accuracy
No. 1	89.09%
No. 2	85.45%
No. 3	87.27%
No. 4	87.27%
No. 5	85.45%
No. 6	89.09%

Table S3. Classification of Braille code. Decision boundaries of SA device's synaptic current for classifying Braille numbers ranging from 0 to 9. The mean firing rate of SA spikes was also used as additional classification criteria for improving accuracy.

**Braille Number**	**Decision boundaries (synaptic response, μA)**	
	**Low**	**High**
"0"	21.96	23.62
"1"	0.00	2.19
"2"	2.19	8.73
"3"	8.73	15.43
"4"	20.34	21.96
"5"	15.43	16.13
"6"	18.06	20.34
"7"	30.79	42.00
"8"	23.62	30.79
"9"	16.13	18.06

Table S4. Relationship between the pairwise spike correlation coefficient (pairwise FA spikes) and the synaptic current (FA device) when detecting different ridge patterns.

Type of ridge patterns	FA spikes' correlation coefficient	FA Device's output (μA)
Parallel	0.936	13.21
Oblique	−0.036	6.11
Crisscross	−0.042	7.37
Circular	0.268	4.91

Table S5. Comparison between different biomimetic mechano- and magneto-sensory systems in terms of fabrication, sensor structure, signal encoding, sensory functions, and neuronal characteristics. SA: slowly adaptive; FA: fast adaptive; FFT: fast Fourier transform; STFT: short-time Fourier transform.

**Mechano- / magneto-sensory system**	**Fabrication method**	**Sensor structure**	**Signal encoding**	**Sensory functions**	**Neuronal / biological characteristics**
Seamless on-skin and implantable sensor [15]	Solution-processing, patterning	Skin-like ultra-thin film	Digital signal	Mechano-perception (pressure, strain)	Implantable bio-interface
Artificial neural tactile sensing system [16]	Solution-processing, patterning	Skin-like multilayer with fingerprints	Spike signal; SA and FA encoding	Mechano-perception (vibration, pressure)	Implantable bio-interface
Three-dimensional piezoelectric polymer microsystems [17]	Solution-processing, dry etching, buckling process	3D serpentine mesostructure	Digital signal (FFT transform)	Mechano-perception (vibration, pressure, bend/stretch)	—
Monolithically integrated, low-voltage, soft e-skin [18]	Solution-processing, patterning, molding	Skin-like micro-structured array	Spike signal; frequency encoding	Mechano-(pressure), thermal perception	Neuromorphic sensorimotor loop (device level); implantable bio-interface
Electronic-skin compasses [19]	E-beam evaporation	Skin-like multilayer	—	Magneto-perception	—
Imperceptible magnetic sensor matrix system [20]	Photo-lithography, magnetron sputtering	Skin-like active matrix	Digital signal (acquisition, addressing, conditioning)	Magneto-perception with field mapping	—
Sensitive pressure sensors integrated with 2D semi-conductor transistors [21]	E-beam process, ALD, molding	Skin-like micro-structured multilayer	Digital signal (STFT transform)	Mechano-perception (pressure, acoustic vibration) with force mapping	—
Neuro-inspired artificial peripheral nervous system [22]	Solution-processing, molding	Skin-like heterogeneous sensor array	Analog spike signal; SA and FA coding	Mechano-(pressure, vibration), thermal perception with spatial mapping	Neuromorphic sensation (circuit-level)
Highly sensitive electronic whisker array [23, 24]	Painting, printing, laser fabrication	Whisker-like resistive sensor array	Digital signal (multiplex reading)	Mechano-(strain), thermal perception with spatial mapping	—
Biomimetic hairy whiskers [25]	Etching, cutting, spray coating	Whisker-like triboelectric sensor	Digital signal (acquisition, conversion, transmission)	Magneto-perception (texture, contact)	—

Insect-inspired electronic antennae (this work)	Solution processing, laser patterning	Insect-antennae-like 3D structure	Spike signal; SA and FA spatiotemporal coding	Mechano- (vibration and contact), magneto-perception	Labeled-line sensory pathway; neuromorphic perception (device-level)

Table S6. Benchmarking our neuromorphic antennal sensory system against the state-of-the-art artificial sensory systems (visual) in the aspect of bio-inspired and insect-inspired perception.

**Bio-inspired / insect-inspired sensory system**	**Sensory modality**	**Structural characteristics**	**Functional characteristics**	**Sensory processing**	**Applications**
Bioinspired vision sensor array with optoelectronic graded neurons [26]	Visual (inspired by flying insect)	Planar structure on Si (fabricated by CVD, lithography)	Motion perception, spatiotemporal information encoding/fusing, temporal summation	Photo-transistor (device-level)	Motion detection, movement recognition
Memristor-based biomimetic compound eye [27]	Visual (inspired by locust's compound eye)	Hemispherical structure on PDMS (fabricated by sputtering, spin coating)	Wide field-of-view (FoV) detection, looming detection	Memristor (device-level)	Machine vision, collision avoidance
Bioinspired vision sensors [28]	Visual (inspired by retina)	Planar structure on Si (fabricated by CVD, lithography)	Scotopic / photopic adaptation	Photo-transistor (device-level)	Image recognition
Vertically integrated spiking cone photoreceptor array [29]	Visual (inspired by drosophila's cone photoreceptor)	Planar structure on Si (fabricated by sputtering)	Spike encoding, light sensing	Memristor (device-level)	Color perception
Biomimetic eye with a hemispherical perovskite nanowire array retina [30]	Visual (inspired by eye)	Hemispherical structure on PDMS (fabricated by electro-deposition, etching)	Image-sensing	Computer	Machine vision
Amphibious artificial vision system with a panoramic visual field [31]	Visual (inspired by crab's compound eye)	Spherical structure on plastic (fabricated by molding, laser ablation, lithography, dry etching)	Amphibious imaging, panoramic imaging	Computer	Panoramic motion detection, obstacle avoidance
Artificial flexible visual memory system [32]	Visual (inspired by retina)	Planar structure on plastic (fabricated by direct printing, deposition)	Visual memory, light detection	Memristor (device-level)	Image processing
Neuromorphic antennal sensory system (this work)	Tactile and magnetic (inspired by insect antennae)	3D structure on plastic (fabricated by solution processing, laser patterning)	Vibrotactile and magneto perception, spatiotemporal recognition, sensory memory	Synaptic transistor (device-level)	Profile / texture / material classifications, robotic navigation, touchless interface

Supplementary References:

1. Derby N, Olbert S. Cylindrical magnets and ideal solenoids. *American Journal of Physics* **78**, 229-235 (2010).
2. Jung KS, Baek YS. Contact-free moving-magnet type of micropositioner with optimized specification. *IEEE transactions on magnetics* **38**, 1539-1548 (2002).
3. Tchumatchenko T, Geisel T, Volgushev M, Wolf F. Spike correlations–what can they tell about synchrony? *Frontiers in Neuroscience* **5**, 68 (2011).
4. Ren Y*, et al.* Gate-tunable synaptic plasticity through controlled polarity of charge trapping in fullerene composites. *Advanced Functional Materials* **28**, 1805599 (2018).
5. Cho JH*, et al.* Printable ion-gel gate dielectrics for low-voltage polymer thin-film transistors on plastic. *Nature Materials* **7**, 900-906 (2008).
6. Wang J*, et al.* A scalable artificial neuron based on ultrathin two-dimensional titanium oxide. *ACS nano* **15**, 15123-15131 (2021).
7. Kumar D, Li H, Das UK, Syed AM, El-Atab N. Flexible Solution-Processable Black-Phosphorus-Based Optoelectronic Memristive Synapses for Neuromorphic Computing and Artificial Visual Perception Applications. *Advanced Materials* **35**, 2300446 (2023).
8. Bessonov AA, Kirikova MN, Petukhov DI, Allen M, Ryhänen T, Bailey MJ. Layered memristive and memcapacitive switches for printable electronics. *Nature Materials* **14**, 199-204 (2015).
9. Liang K*, et al.* Fully printed optoelectronic synaptic transistors based on quantum dot–metal oxide semiconductor heterojunctions. *ACS nano* **16**, 8651-8661 (2022).
10. Ham S*, et al.* One-dimensional organic artificial multi-synapses enabling electronic textile neural network for wearable neuromorphic applications. *Science Advances* **6**, eaba1178 (2020).
11. Wang Y*, et al.* Memristor-based biomimetic compound eye for real-time collision detection. *Nature Communications* **12**, 5979 (2021).
12. Wan H, Cao Y, Lo L-W, Zhao J, Sepulveda N, Wang C. Flexible carbon nanotube synaptic transistor for neurological electronic skin applications. *ACS nano* **14**, 10402-10412 (2020).
13. Shim H*, et al.* Artificial neuromorphic cognitive skins based on distributed biaxially stretchable elastomeric synaptic transistors. *Proceedings of the National Academy of Sciences* **119**, e2204852119 (2022).
14. Kim Y*, et al.* A bioinspired flexible organic artificial afferent nerve. *Science* **360**, 998-1003 (2018).
15. Jiang Z*, et al.* A 1.3-micrometre-thick elastic conductor for seamless on-skin and implantable sensors. *Nature Electronics* **5**, 784-793 (2022).
16. Chun S*, et al.* An artificial neural tactile sensing system. *Nature Electronics* **4**, 429-438 (2021).
17. Han M*, et al.* Three-dimensional piezoelectric polymer microsystems for vibrational energy harvesting, robotic interfaces and biomedical implants. *Nature Electronics* **2**, 26-35 (2019).
18. Wang W*, et al.* Neuromorphic sensorimotor loop embodied by monolithically integrated, low-voltage, soft e-skin. *Science* **380**, 735-742 (2023).
19. Cañón Bermúdez GS, Fuchs H, Bischoff L, Fassbender J, Makarov D. Electronic-skin compasses for geomagnetic field-driven artificial magnetoreception and interactive electronics. *Nature Electronics* **1**, 589-595 (2018).
20. Kondo M*, et al.* Imperceptible magnetic sensor matrix system integrated with organic driver and amplifier circuits. *Science Advances* **6**, eaay6094 (2020).
21. Huang Y-C*, et al.* Sensitive pressure sensors based on conductive microstructured air-gap gates and two-dimensional semiconductor transistors. *Nature Electronics* **3**, 59-69 (2020).
22. Lee WW*, et al.* A neuro-inspired artificial peripheral nervous system for scalable electronic skins. *Science Robotics* **4**, eaax2198 (2019).
23. Takei K, Yu Z, Zheng M, Ota H, Takahashi T, Javey A. Highly sensitive electronic whiskers based on patterned carbon nanotube and silver nanoparticle composite films. *Proceedings of the National Academy of Sciences* **111**, 1703-1707 (2014).
24. Harada S, Honda W, Arie T, Akita S, Takei K. Fully printed, highly sensitive multifunctional artificial electronic whisker arrays integrated with strain and temperature sensors. *ACS nano* **8**, 3921-3927 (2014).
25. An J*, et al.* Biomimetic hairy whiskers for robotic skin tactility. *Advanced Materials* **33**, 2101891 (2021).
26. Chen J, *et al.* Optoelectronic graded neurons for bioinspired in-sensor motion perception. *Nature Nanotechnology* **18**, 882–888 (2023).

27. Wang Y, *et al.* Memristor-based biomimetic compound eye for real-time collision detection. *Nature Communications* **12**, 5979 (2021).
28. Liao F, *et al.* Bioinspired in-sensor visual adaptation for accurate perception. *Nature Electronics* **5**, 84–91 (2022).
29. Wang X, *et al.* Vertically integrated spiking cone photoreceptor arrays for color perception. *Nature Communications* **14**, 3444 (2023).
30. Gu L, *et al.* A biomimetic eye with a hemispherical perovskite nanowire array retina. *Nature* **581**, 278–282 (2020).
31. Lee M, *et al.* An amphibious artificial vision system with a panoramic visual field. *Nature Electronics* **5**, 452–459 (2022).
32. Chen S, Lou Z, Chen D, Shen G. An artificial flexible visual memory system based on an UV-motivated memristor. *Advanced Materials* **30**, 1705400 (2018).
33. Wan X, Cong H, Jiang G, Liang X, Liu L, He H. A review on PVDF nanofibers in textiles for flexible piezoelectric sensors. *ACS Applied Nano Materials* 6, 1522-1540 (2023).
34. Chen F, *et al.* Recent progress in artificial synaptic devices: materials, processing and applications. *Journal of Materials Chemistry C* 9, 8372-8394 (2021).
35. Chorsi MT, *et al.* Piezoelectric biomaterials for sensors and actuators. *Advanced Materials* 31, 1802084 (2019).
36. Lee JH, Cho KH, Cho K. Emerging trends in soft electronics: integrating machine intelligence with soft acoustic/vibration sensors. *Advanced Materials* 35, 2209673 (2023).
37. Lu L, Ding W, Liu J, Yang B. Flexible PVDF based piezoelectric nanogenerators. *Nano Energy* 78, 105251 (2020).
38. Wang D, Zhao S, Yin R, Li L, Lou Z, Shen G. Recent advanced applications of ion-gel in ionic-gated transistor. *Npj Flexible Electronics* 5, 13 (2021).
39. Zhu J, Zhang T, Yang Y, Huang R. A comprehensive review on emerging artificial neuromorphic devices. *Applied Physics Reviews* 7, 011312 (2020).
